# Inherited duplications of *PPP2R3B* predispose to nevi and melanoma via a *C21orf91*-driven proliferative phenotype

**DOI:** 10.1038/s41436-021-01204-y

**Published:** 2021-06-18

**Authors:** Satyamaanasa Polubothu, Davide Zecchin, Lara Al-Olabi, Daniël A. Lionarons, Mark Harland, Stuart Horswell, Anna C. Thomas, Lilian Hunt, Nathan Wlodarchak, Paula Aguilera, Sarah Brand, Dale Bryant, Cristina Carrera, Hui Chen, Greg Elgar, Catherine A. Harwood, Michael Howell, Lionel Larue, Sam Loughlin, Jeff MacDonald, Josep Malvehy, Sara Martin Barberan, Vanessa Martins da Silva, Miriam Molina, Deborah Morrogh, Dale Moulding, Jérémie Nsengimana, Alan Pittman, Joan-Anton Puig-Butillé, Kiran Parmar, Neil J. Sebire, Stephen Scherer, Paulina Stadnik, Philip Stanier, Gemma Tell, Regula Waelchli, Mehdi Zarrei, Susana Puig, Véronique Bataille, Yongna Xing, Eugene Healy, Gudrun E. Moore, Wei-Li Di, Julia Newton-Bishop, Julian Downward, Veronica A. Kinsler

**Affiliations:** 1 Mosaicism and Precision Medicine Laboratory, Francis Crick Institute, London, UK; 2 Genetics and Genomic Medicine, UCL GOS Institute of Child Health, London, UK; 3 Paediatric Dermatology, Great Ormond Street Hospital for Children, London, UK; 4 Oncogene Biology Laboratory, Francis Crick Institute, London, UK; 5 Section of Epidemiology and Biostatistics, Leeds Institute of Cancer and Pathology, Cancer Research UK Clinical Centre at Leeds, St James’s University Hospital, Leeds, UK; 6 Bioinformatics and Biostatistics, Francis Crick Institute, London, UK; 7 Advanced Sequencing Facility, Francis Crick Institute, London, UK; 8 McArdle Laboratory, Department of Oncology, University of Wisconsin–Madison, School of Medicine and Public Health, Madison, WI USA; 9 Department of Dermatology, Hospital Clínic de Barcelona (Melanoma Unit), University of Barcelona, IDIBAPS, Barcelona & CIBERER, Barcelona, Spain; 10 Centre for Cell Biology and Cutaneous Research, Blizzard Institute, Barts, London, UK; 11 High Throughput Screening Facility, Francis Crick Institute, London, UK; 12 Centre de Recherche, Developmental Genetics of Melanocytes, Institut Curie, Orsay, France; 13 North East Thames Regional Genetics Laboratory Service, Great Ormond Street Hospital for Children NHS Foundation Trust, London, UK; 14 The Centre for Applied Genomics and Program in Genetics and Genome Biology, The Hospital for Sick Children, Toronto ON, Canada; 15 Bioinformatics, St George’s University of London, London, UK; 16 Department of Twin Research and Genetic Epidemiology, King’s College London, South Wing Block D, London, UK; 17 Department of Histopathology, Great Ormond Street Hospital for Children, London, UK; 18 Department of Dermatology, University Hospital Southampton NHS Foundation Trust, Southampton, UK; 19 Infection, Immunity and Inflammation Programme, Immunobiology Section, UCL GOS Institute of Child Health, London, UK

## Abstract

**Purpose:**

Much of the heredity of melanoma remains unexplained. We sought predisposing germline copy-number variants using a rare disease approach.

**Methods:**

Whole-genome copy-number findings in patients with melanoma predisposition syndrome congenital melanocytic nevus were extrapolated to a sporadic melanoma cohort. Functional effects of duplications in *PPP2R3B* were investigated using immunohistochemistry, transcriptomics, and stable inducible cellular models, themselves characterized using RNAseq, quantitative real-time polymerase chain reaction (qRT-PCR), reverse phase protein arrays, immunoblotting, RNA interference, immunocytochemistry, proliferation, and migration assays.

**Results:**

We identify here a previously unreported genetic susceptibility to melanoma and melanocytic nevi, familial duplications of gene *PPP2R3B*. This encodes PR70, a regulatory unit of critical phosphatase PP2A. Duplications increase expression of PR70 in human nevus, and increased expression in melanoma tissue correlates with survival via a nonimmunological mechanism. *PPP2R3B* overexpression induces pigment cell switching toward proliferation and away from migration. Importantly, this is independent of the known microphthalmia-associated transcription factor (MITF)-controlled switch, instead driven by *C21orf91*. Finally, C21orf91 is demonstrated to be downstream of MITF as well as PR70.

**Conclusion:**

This work confirms the power of a rare disease approach, identifying a previously unreported copy-number change predisposing to melanocytic neoplasia, and discovers *C21orf91* as a potentially targetable hub in the control of phenotype switching.

## INTRODUCTION

Melanoma (CMM [MIM 155600]) remains a major cause of morbidity and mortality. The majority of the heredity of melanoma remains unexplained, with germline variants in *CDKN2A* in 2% of cases the commonest known genetic predisposer.^1^ Identification of new susceptibility genes is desirable to improve understanding of the condition at molecular level, with a view to better therapeutic options. We sought to identify novel susceptibility loci for melanocytic neoplasia, using a rare disorder approach.

Congenital melanocytic nevi (CMN [MIM 137550]) is a rare mosaic disorder of large and multiple moles, which predisposes affected individuals to melanoma. It is a valuable UV-independent genetic model for the development of melanoma, with causative somatic pathogenic variants in *NRAS* in 70%,^2,3^ and *BRAF* in 7%,^3,4^ with the remainder as yet unknown. Despite the sporadic somatic nature of the disease, one-third of cases have a first or second degree family history of CMN in the largest published cohort,^5,6^ suggesting germline susceptibility to *NRAS*/*BRAF* somatic pathogenic variant in affected families, including the already established variants in *MC1R.*^6^ We hypothesized that new predisposing copy-number variants found via this rare disorder could also predispose to melanoma in the normal population.

Germline copy number in the CMN cohort was measured using an unbiased whole genome approach, and relevant findings validated in an adult melanoma cohort. Previously unreported duplications of gene *PPP2R3B* were discovered in both cohorts, at a frequency comparable to that of pathogenic *CDKN2A* variants. Extensive modeling of the biology of *PPP2R3B* overexpression demonstrated promotion of proliferation and reduction of migration in melanoma cells. The balance between proliferation and migration/invasion is known as pigment cell phenotype switching, and its regulation is recognized as critical in melanoma progression and treatment.^7,8^ This process was, surprisingly, independent of the key regulator of pigment cell phenotype switching, microphthalmia-associated transcription factor (MITF).^8,9^ Whole-genome RNAseq instead revealed that *PPP2R3B* overexpression drives pigment phenotype switching via largely uncharacterized gene *C21orf91*. Moreover, MITF-driven proliferation in melanoma cells is rescuable by *C21orf91* knockdown, indicating that C21orf91 is downstream of both PR70 and MITF in our in vitro system, and as such is a potentially targetable hub in melanoma.

## MATERIALS AND METHODS

For methods of patient recruitment, ethical approvals, immunohistochemistry, immunocytochemistry, quantitative real-time polymerase chain reaction (qRT-PCR), western blotting, sample preparation, and all methods relating to supplementary results figures, please see Supplementary Material.

### Array CGH

Whole-genome array comparative genomic hybridization (CGH) was performed as per the manufacturer’s instructions on 24 germline DNA samples from the CMN cohort, using Roche Nimblegen 135K oligonucleotide arrays and sex-matched commercial pooled controls. 1–3 μg of patient DNA and control DNA (Megapool reference DNA, male: EA-100M, female: EA-100F, Kreatech, The Netherlands) was labeled using NimbleGen Dual-Color DNA Labeling Kit and hybridized to the oligonucleotide array using the NimbleGen Hybridization System. Two-color array scanning was performed using a Molecular Devices GenePix 4400A (Molecular Devices, Sunnyvale, CA, USA) at a resolution of 2.5 microns. Data were extracted using Deva software (NimbleGen), and analyzed using InfoQuant CGHFusion (version 5.7.0–6.1.0) or later Chromosome Analysis Suite 4.0 (ChAS 4.0, Thermo Fisher Scientific). Abnormal copy number was called as per diagnostic facility criteria: at least 3 consecutive probe points above or below the zero line, with an average ratio of difference in fluorescence at least +/−0.4 in those points, and excluding areas where copy-number variants had already been reported.

### Targeted next-generation sequencing panel

A SureSelect targeted panel (Agilent Technologies, UK) was designed to capture the whole genomic region encompassing the telomeric 4 genes on the pseudoautosomal region of X and Y chromosomes, chrX:198061-607558 chrY:148061-557558 (hg19/GRCh37). Library preparation was by SureSelectXT kit under manufacturer’s instructions (Agilent Technologies, UK), and sequencing on NextSeq instrument 500/550, read length of 2 × 150 bp (Illumina, USA); Leeds melanoma samples (*n* = 168) and GOSH CMN samples (*n* = 5). BAM files were inputted to DeepTools MultiBamSummary using the PPP2R3B_Moderately_Stringent_1_covered.bed probe coordinates file. Coverage across probe regions within hg19 coordinates were extracted and averaged. Three control samples were used to create “normal expected” coverage ratios of the genes compared to *SHOX*. All samples were normalized compared to these ratios and R studio was used to visualize and calculate gene coverage data across all samples.

### Generation of stable inducible *PPP2R3B* cell lines

SKMEL2 and SKMEL30 melanoma cell lines carry variants affecting codon 61 of NRAS, and were cultured as per manufacturer’s instructions. Normal *PPP2R3B* copy number in both cell lines was verified by next-generation sequencing (NGS). Human *myc-*FLAG tagged *PPP2R3B* ORF clone from Origene (RC222908) was linearized and the insert DNA amplified using modified primers generating an N-terminal Myc tag. The In-Fusion® HD Cloning system (Takara, 638909) was used to allow directional cloning of the *PPP2R3B* insert into the AgeI-MluI site of the lentiviral vector pTRIPZ (GE Healthcare) resulting in the final *PPP2R3B* (tet-ON) construct, without the TurboRFP or shRNAmir-related elements of the parental pTRIPZ plasmid. Transduction of HEK 293T cells with pTRIPZ-*PPP2R3B* in addition to psPAX2 and pMD2.G lentiviral plasmids using Lipofectamine 2000™ generated lentiviral particles used to infect SKMEL2 or SKMEL30 target cells using polybrene to enhance efficiency. Stable cell lines were selected using puromycin.

### Reverse phase protein array

Protein samples were diluted to 1.5 µg/µl and submitted to MD Anderson, Core Facility. Reported intensity values were log transformed to approximate normality and comparisons were performed using an unpaired *t*-test.

### RNAseq

RNA integrity was assessed using a Bioanalyser (Agilent). Library preparation using KAPA messenger RNA (mRNA) HyperPrep Kit (Roche) was automated using the Hamilton robot, and sequenced using a NextSeq 500 (Illumina, San Diego, CA, USA) with a 43-bp paired-end run. Data were trimmed for 3’ adapter sequences using Cutadapt 1.9.1, after which they were aligned to the Ensembl GRCh38 release 86 human transcriptome using STAR 2.5.2a. Individual lane level replicates were merged using Samtools 1.8, raw gene counts estimated using RSEM 1.3.0, and normalization and differential expression called using DESeq2. A corrected *p* value of <0.05 was deemed significant. Pathway analyses based on genes reported in the various analyses were performed using Metacore (Clarivate Analytics).

### Proliferation assays

#### WST1 proliferation assay

SKMEL2-pTRIPZ-PPP2R3B and SKMEL30-pTRIPZ-PPP2R3B cells were seeded into a 96-well plate at a density of 1 × 10^4^ cells/well. *PPP2R3B* overexpression was induced alongside uninduced controls. Plates were incubated for 48 hours at 37 °C, prior to addition of WST-1 reagent. Plates were incubated in the dark at 37 °C for two hours. Absorbance was read by spectrophotometer at 450 nm and 620 nm, adjusted for absorbance of blank media and of WST1 dye (620 nm), and averaged across replicates (mean + SD). Significance was calculated by Student’s *t*-test.

#### BrDU proliferation assay

BrdU Cell Proliferation ELISA Kit, (Abcam, ab126556) was used as per the manufacturer’s instructions. SKMEL2-pTRIPZ-PPP2R3B and SKMEL30-pTRIPZ-PPP2R3B cells were seeded into a 96-well plate at a density of 2 × 10^5^ cells/well, *PPP2R3B* overexpression induced alongside uninduced and suggested assay controls. Absorbance was averaged across replicates (mean + SD) and significance calculated by Student’s *t*-test.

#### *PPP2R3B* overexpression IncuCyte® Cell Count Proliferation Assay

SKMEL2-pTRIPZ-PPP2R3B and SKMEL30-pTRIPZ-PPP2R3B cells were seeded into a 96-well ImageLock plate at a density of 1 × 10^4^ cells/well. *PPP2R3B* overexpression was induced alongside uninduced control, a total of 12 replicates per condition. IncuCyte**®** live-cell analysis acquired 10× phase contrast images at a scanning interval of 60 minutes for 5 days, measuring percentage confluence. Confluence was averaged across replicates (mean + SD) and significance was calculated by Student’s *t*-test.

#### *C21orf91* knockdown

The efficacy of three *C21orf91* small interfering RNAs (siRNAs) (Origene, SR310041) was assessed in cell line SKMEL2 at concentrations of 1 nM, 5 nM, 10 nM, and 25 nM, and knockdown confirmed by qRT-PCR and western blotting (Fig. S7). SKMEL2-pTRIPZ-PPP2R3B was seeded into a 96-well ImageLock plate at a density of 1 × 10^4^ cells/well, one plate for the proliferation and one for the scratch wound assay. *PPP2R3B* overexpression was induced alongside uninduced controls, 12 replicates per condition. Cells were transfected with Lipofectamine™ RNAiMAX using one or two *C21orf91* siRNAs or a scrambled siRNA (Origene, SR310041) at a concentration of 10 nm. For the scratch wound assay WoundMaker™ created a scratch in each well. Plates were washed with PBS and fresh media added. IncuCyte® live-cell analysis system acquired 10× phase contrast images at a scanning interval of 60 minutes for 5 days, for the proliferation assay and for 3 days for the scratch wound assay. Confluence was averaged across replicates (mean + SD) and significance calculated by Student’s *t*-test. Relative wound confluence was averaged across replicates (mean + SD) and significance calculated by Student’s *t-*test.

#### *MITF* knockdown and overexpression

Cells were transfected with Lipofectamine™ RNAiMAX using two *MITF* siRNAs (siMITF 1: AAAGCAGTACCTTTCTACCAC; siMITF 2: TGGCTATGCTTACGCTTAA^10^ or scrambled siRNA at a concentration of 25 nm and knockdown was confirmed by qRT-PCR.

*MITF* overexpression was obtained by transiently transfecting cells with Lipofectamine 2000 and pCMV-TAG4A-MITF-M-wt plasmid (Addgene cat. 31151). Empty control vector was obtained by excision of *MITF* coding sequence from pCMV-TAG4A-MITF-M-wt plasmid (EcoRI and MfeI combined digestion) and ligation of compatible ends. Overexpression was confirmed through comparison by qRT-PCR of MITF-transfected and control vector-transfected cells.

#### Scratch wound assay

SKMEL2-pTRIPZ-PPP2R3B and SKMEL30-pTRIPZ-PPP2R3B cells were seeded into a 96-well ImageLock plate at a density of 1 × 10^5^ cells/well. *PPP2R3B* overexpression was induced alongside uninduced controls, 12 replicates per condition, and plates incubated at 37 degrees until all wells were confluent. WoundMaker™ created a scratch in each well. Plates were washed with PBS and fresh media added. IncuCyte**®** live-cell analysis system acquired 10× phase contrast images at scanning intervals of 60 minutes. Relative wound confluence was averaged across replicates (mean + SD) and significance calculated by Student’s *t*-test.

#### Leeds Melanoma Cohort: transcriptomic data

Whole transcriptomes were derived from 703 formalin-fixed, paraffin-embedded (FFPE) primary cutaneous melanomas from the Leeds Melanoma Cohort^11^ (median follow-up 7.5 years) using the Illumina DASL HT12.4 array. Kaplan–Meier survival analysis used melanoma-specific survival (MSS), after correction for known confounding factors age, sex, American Joint Committee for Cancer (AJCC) stage, vascular invasion, site, *BRAF/NRAS* pathogenic variant status, and tumor invading lymphocytes (TILs).

## RESULTS

### Germline duplications involving *PPP2R3B* are found at increased frequency in individuals with melanocytic neoplasia

**Fig. 1:**
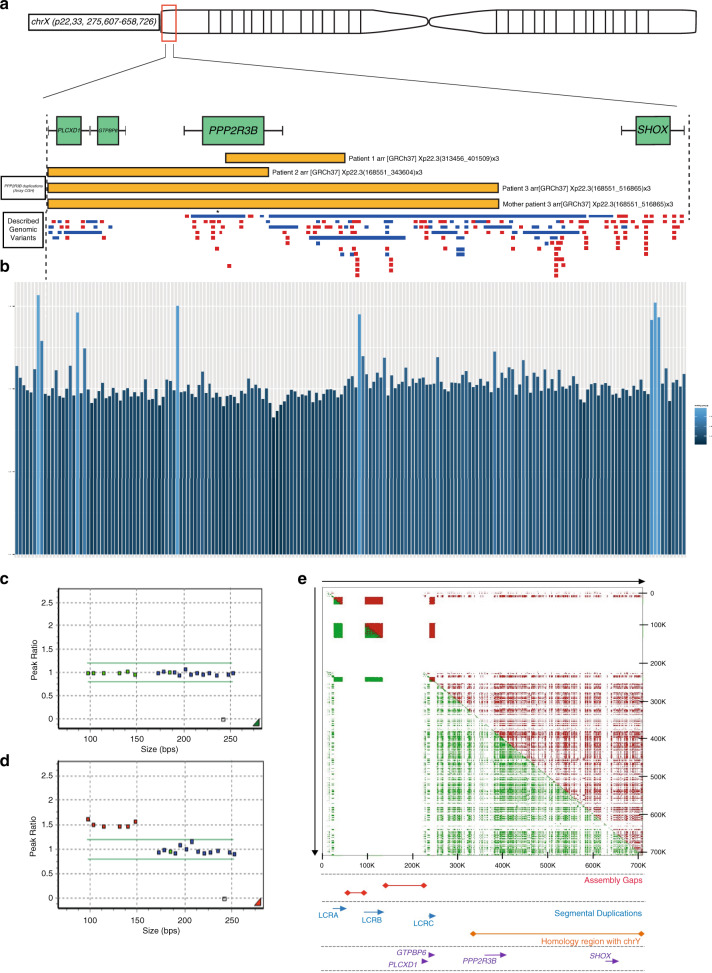
Germline duplications involving PPP2R3B are found at increased frequency in individuals with melanocytic neoplasia. (**a**) Schematic of Xp22.33 demonstrating the location of three novel duplications (yellow) found in 24 congenital melanocytic nevi (CMN) patients using whole-genome array comparative genomic hybridization (CGH) of leukocyte DNA, with one identical parental duplication demonstrating inheritance. Previously described copy-number variants in that region are shown below, duplications in blue, deletions in red, with each bar representing a single publication. The publication representing a duplication involving *PPP2R3B* described a single variant in a cohort of approximately 36,000 (asterisk; see text for details), confirming that the CMN duplications are rare in the normal population. (**b**) *PPP2R3B* duplications in a UK nonsyndromic melanoma cohort (4 duplications in nonselected cohort *n* = 168), and CMN cohort (3 duplications in known preselected cohort *n* = 5) shown by targeted next-generation sequencing (NGS) of *PPP2R3B*, in addition to the two telomeric genes (*GTPBP6* and *PLCXD1*) and the next centromeric gene (*SHOX*). Data represent the ratio of corrected read depth (see text for details) across the whole of *PPP2R3B* with respect to the ratio across the whole of *SHOX*. Each bar represents an individual patient. *PPP2R3B* duplications called are shown in light blue: validation of the array CGH findings in the three CMN patients are clustered to the right of the figure, and new duplications in the melanoma cohort in the rest of the figure (*n* = 4, 2.4%). Validation of PPP2R3B duplications detected by array CGH. Custom-designed multiplex ligation-dependent probe amplification (MLPA) ratio plots validating copy-number measurement of *PPP2R3B* (4 probes) and the two telomeric genes *GTPBP6* and *PLCXD1* (one probe each), to the left of each figure and less than 150 bp in length; control probes of greater than 160 kb targeting genes of known normal copy number across the genome are shown to the right at greater than 150 bp size. A representative example of normal copy number for all genes (**c**), and of a duplication of *PPP2R3B* and *GTPBP6* and *PLCXD1* in a CMN patient (red dots) (**d**). While this method was able to validate the array CGH findings, it was not as robust as the targeted NGS panel for novel discovery of copy-number changes, likely due to the repetitive, GC-rich, and polymorphic nature of the region studied. Low-copy repeats at Xp22.33. (**e**) The upper panel depicts a regional similarity search across Xp22.33 with YASS software (http://bioinfo.cristal.univ-lille.fr/yass/index.php) both forward (green) and backward (red) revealing three segmental duplications (LCRA, LCRB, and LCRC) 5’ of *PPP2R3B* and a high density of SINE and LINE repeats. No segmental duplications are detected 3’ to *PPP2R3B* before *SHOX*. The assembly gaps (red), local genes (purple), and the homology region (orange) with the Y chromosome are indicated.

Using whole-genome array CGH of leukocyte DNA, duplications of Xpter were identified in 3/24 (12.5%) randomly selected patients with CMN, where only gene *PPP2R3B* was common to all three (Fig. 1a). These three patients had causative postzygotic pathogenic variants affecting codon 61 of *NRAS* in two cases, and no identified causative pathogenic variants (non-*NRAS*, non-*BRAF*) in the third. No other undescribed copy-number variant was seen in more than one patient. Control data from pediatric patients with other phenotypes from the same diagnostic testing facility identified duplications of this gene (with or without involvement of the two telomeric genes but not extending centromeric) in only 13/4,800, or 0.271%. Population data from normal individuals from the Database of Genomic Variants^12^ identified similar duplications in only 1/36,000, or 0.003%,^13^ and 0.5% in nearly 7,000 individuals in the MSSNG autism study,^14,15^ with no difference between cases and controls (personal communication). High-depth targeted NGS was eventually selected as the most robust readout for copy number in this repetitive GC-rich telomeric region. NGS confirmed the CMN array findings and on screening leukocyte DNA from an adult sporadic melanoma cohort, identified the same germline duplications in 4/168 (2.4%) (all *BRAF* p.V600E), (Fig. 1b), demonstrating that this copy-number variant is enriched in populations with melanocytic neoplasia. Custom-designed multiple ligation-dependent probe amplification (MLPA, MRC Holland) validated the array CGH findings and suggested the prevalence of duplications involving *PPP2R3B* to be 5% of the total cohort of 125 individuals with CMN (Fig. 1c, d), but was not as robust as CGH or NGS for duplication discovery. Regional similarity search across Xp22.33 revealed three segmental duplications 5’ of *PPP2R3B*, and a high density of SINE and LINE repeats, but no segmental duplications between *PPP2R3B* and *SHOX* (Fig. 1e). Sanger sequencing of leukocyte DNA from 48 CMN patients and 48 normal controls did not detect any unreported variants or haplotype differences (data not shown).

### Germline duplications of *PPP2R3B* lead to increased expression of PR70 in congenital melanocytic nevi

**Fig. 2:**
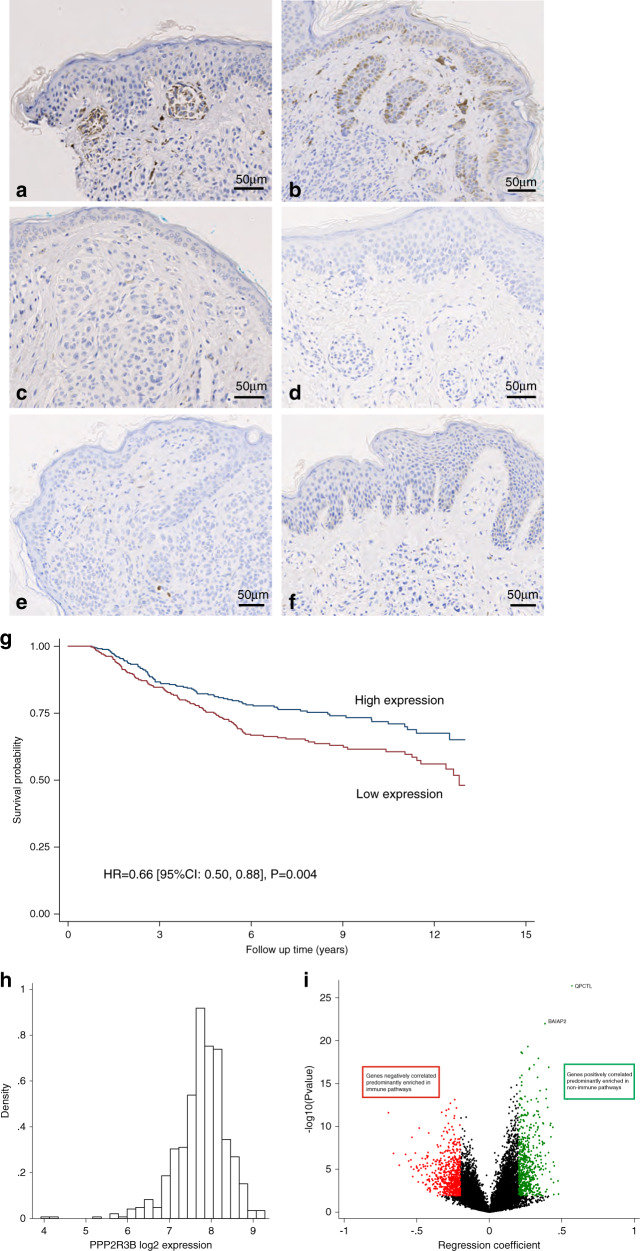
Germline duplications of *PPP2R3B* lead to increased expression of protein product PR70 in congenital melanocytic nevi (CMN) tissue, compared to that of normal copy-number controls. (**a**, **b**) Immunohistochemical staining of formalin fixed paraffin embedded (FFPE) CMN tissue demonstrates moderate intensity PR70 staining throughout the cytoplasm of nevus cells in two patients where tissue was available with a confirmed germline *PPP2R3B* duplication and (**c**–**f**). Negative PR70 staining in four patients with confirmed normal copy number of *PPP2R3B*. Stained sections were assessed by two independent blinded assessors and assigned a score of 1–3, based on the intensity of staining observed and scores averaged. Scores were as follows: a = 3, b = 2.5, c = 0, d = 0, e = 0, and f = 0. Increased *PPP2R3B* expression in melanoma tissue is correlated with improved melanoma specific survival. (**g**) Kaplan–Meier curve generated from transcriptomic data from 703 FFPE melanoma tumors from the Leeds Melanoma Cohort, hazard ratio (HR) = 0.66, (95% confidence interval [CI] 0.50–0.88), *p* = 0.004. The effect remains significant after adjusting for age, sex, American Joint Committee for Cancer (AJCC) stage, vascular invasion, site, *BRAF/NRAS* pathogen variant status, and tumor invading lymphocytes (TILs). (**h**) Log intensity distribution of PPP2R3B DASL probe (ILMN_1689720) is close to a normal distribution. Improved melanoma specific survival observed with increased expression of *PPP2R3B* appears not to be immune mediated. Tumor expression of *PPP2R3B* correlates with expression of a large number of other genes in the genome: 596 positively correlated at FDR < 0.05 with regression coefficient >0.20; 731 negatively correlated at FDR < 0.05 with a regression coefficient < −0.2. (**i**) The genes positively correlated with *PPP2R3B* are predominantly enriched in nonimmune pathways, consistent with the lack of association between *PPP2R3B* expression and TILs or any specific immune cell score. The genes negatively correlated with PPP2R3B expression are predominantly enriched in immune pathways (Table S1, 2).

Owing to other potential genetic confounders within malignant tissue such as loss of Xp, effects of germline duplication on tissue expression in vivo was visualized by immunohistochemistry in CMN tissue, known to have little somatic copy-number variation.^16^ Germline duplications were clearly associated with increased expression of PR70 in the available CMN tissue on immunohistochemistry compared to controls (Fig. 2a–f).

### Expression of PR70 is significantly associated with prolonged MSS

Using published data of whole-genome transcriptomic profiling of 703 melanomas,^11^ increased tissue expression of *PPP2R3B* was significantly associated with prolonged MSS (Fig. 2g, h). This effect remained significant after correction for known associations with survival, namely age, sex, AJCC stage, vascular invasion, site, *BRAF/NRAS* pathogenic variant status, and TILs. Unlike many known expression-survival associations in melanoma, transcriptomic pathway analysis did not support an immune pathway-mediated effect (Fig. 2i, Tables S3, S4), leading us to look for an alternative mechanism of action of *PPP2R3B* overexpression on melanocytic proliferation.

### Creation of a stable inducible overexpression system to study *PPP2R3B* overexpression

**Fig. 3:**
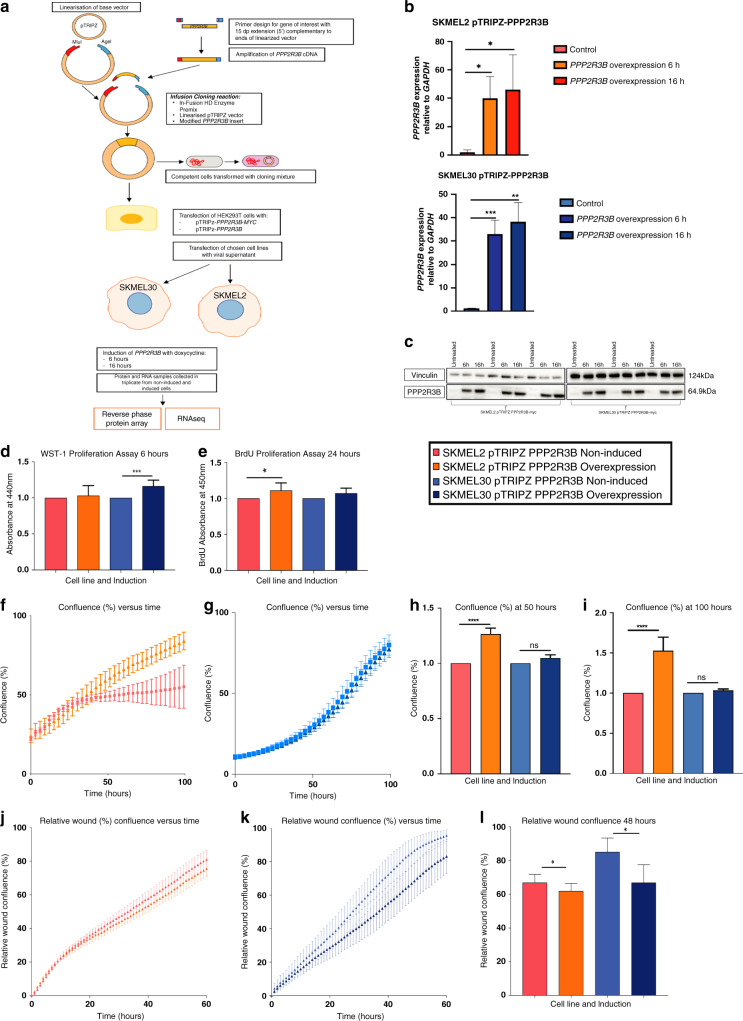
Generation of stable inducible overexpression model for *PPP2R3B* in melanoma cell lines SKMEL2 and SKMEL30. (**a**) Diagrammatic representation of stable inducible *PPP2R3B* overexpression system (SKMEL2-pTRIPZ-PPP2R3B and SKMEL30-pTRIPZ-PPP2R3B), and generation of samples for reverse phase protein arrays (RPPA) and RNA sequencing. Validation of *PPP2R3B* overexpression in samples for reverse phase protein array and RNAseq: (**b**) quantitative real-time polymerase chain reaction (qRT-PCR) demonstrating increased *PPP2R3B* messenger RNA (mRNA) in both induced cell lines at 6 hours and 16 hours (relative fold change in PPP2R3B expression, standardized to GAPDH, mean + SD of samples in quadruplicate), and (**c**) western blot confirming PR70 overexpression in induced cell lines at 6 hours and 16 hours with vinculin loading control. Statistical significance was determined using a Student’s *t*-test (Prism v7.0, Graphpad). Statistically significant values are indicated by a single asterisk (*p* < 0.05), a double asterisk (*p* < 0.01), a triple asterisk (*p* < 0.001), or a quadruple asterisk (*p* < 0.0001). Overexpression of *PPP2R3B* increases proliferation in melanoma cell lines. (**d**) Increased proliferation following *PPP2R3B* overexpression using WST1 proliferation assay in SKMEL2-pTRIPZ-PPP2R3B at 6 hours, (**e**) in SKMEL30-pTRIPZ-PPP2R3B by BrdU assay at 24 hours (mean absorbance of colormetric assay of eight replicates shown with standard deviation), and (**f**, **g**) by IncuCyte® cell count proliferation assay in SKMEL2-pTRIPZ-PPP2R3B and SKMEL30-pTRIPZPPP2R3B respectively over 100 hours, measuring confluence (%) versus time (hours) (mean confluence of eight replicates with standard deviation). (**h**, **i**) Mean confluence in each cell line is shown at timepoints of 50 hours and 100 hours respectively (mean of eight replicates standardized to noninduced cell line shown with standard error). Statistical analysis was performed and depicted as described above in figure legend. Overexpression of *PPP2R3B* decreases cellular migration in melanoma cell lines. (**j**, **k**) Scratch wound assay in SKMEL2-pTRIPZ-PPP2R3B and SKMEL30-pTRIPZ-PPP2R3B respectively leads to decreased relative wound confluence (%) versus time (hours) compared to noninduced controls. (**l**) Mean relative wound confluence in both cell lines shown at 48 hours (mean of eight replicates shown with error bars). Statistical analysis was performed and depicted as described above in figure legend.

The effects and mechanisms of *PPP2R3B* overexpression were modeled in detail by creation of a stable inducible overexpression system in two *NRAS*-mutant melanoma cell lines SKMEL2 and SKMEL30 (Fig. 3a–c). Induction robustly and reproducibly led to *PPP2R3B* mRNA overexpression, and PR70 protein overexpression (antibody validated by CRISPR/Cas9 knockout, Fig. S6).

### *PPP2R3B* overexpression leads to increased cellular proliferation and decreased migration in 2D melanoma cell models

Overall *PPP2R3B* overexpression led to pigment cell phenotype switching. This was measurable via significantly increased cellular proliferation by several alternative established methods, with some variation between cell lines (Fig. 3d–i), and decreased migration in scratch assays coupled to IncuCyte® monitoring (Fig. 3j–l). siRNA knockdown of *PPP2R3B* did not alter proliferation (Fig. S7), in line with the clinical data demonstrating duplications but not deletions in melanocytic neoplasia cohorts.

### *PPP2R3B* overexpression does not significantly alter known melanoma signaling pathway activation

**Fig. 4:**
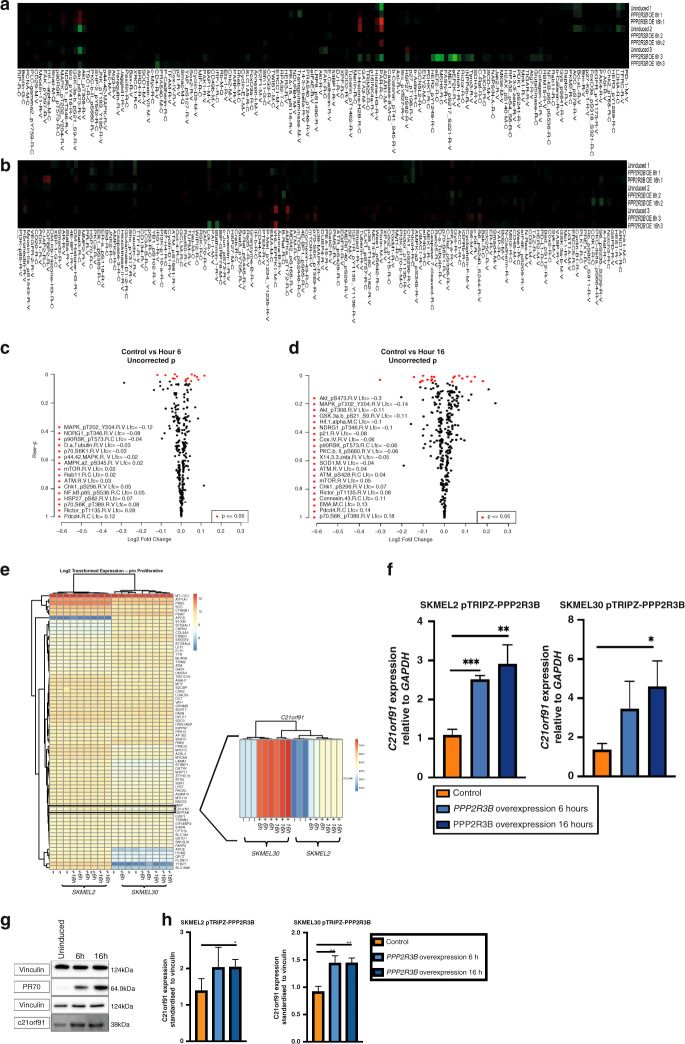
*PPP2R3B* overexpression affects mTOR/p70S6K1 and HIF-1 signaling pathways. (**a**, **b**) Heat map of protein expression observed by reverse phase protein arrays (RPPA) following overexpression of *PPP2R3B* in SKMEL2-pTRIPZ-PPP2R3B and SKMEL30-pTRIPZ-PPP2R3B respectively, demonstrating low background activity as expected from a controlled cellular model. (**c**, **d**) Volcano plots of log fold change in protein expression versus *p* value for differentially expressed proteins common to both cell lines following *PPP2R3B* overexpression at 6 hours and 16 hours respectively. Unadjusted *p* values < 0.05 are shown in red. Raw data is available in the Supplementary material (Table S4). PPP2R3B overexpression leads to significant and sustained rise in expression of gene *C21orf91*. *C21orf91* was the most differentially expressed gene on *PPP2R3B* induction common to both cell lines and at both time points by RNA sequencing (Table S5), other than *PPP2R3B* itself. (**e**) Heat map from pathway signature analysis of RNAseq data at 6 hours and 16 hours, focusing on pro-proliferative anti-invasive melanoma signature genes38, demonstrating increased expression of *C21orf91* following *PPP2R3B* overexpression observed in both cell lines, at 6 and 16 hours. Validation of significantly increased *C21orf91* expression following *PPP2R3B* overexpression at 6 hours and 16 hours in both cell lines, shown by (**f**) quantitative real-time polymerase chain reaction (qRT-PCR) relative fold change in *C21orf91* mRNA levels, samples standardized to GAPDH (mean + SD of samples in quadruplicate) and (**g**, **h**) representative western blot with quantification of fold change of *C21orf91—*samples standardized to vinculin (mean shown with standard deviation of samples in triplicate). Statistical significance was determined using a Student’s *t*-test (Prism v7.0, Graphpad). Statistically significant values are indicated by a single asterisk (*p* < 0.05), a double asterisk (*p* < 0.01), a triple asterisk (*p* < 0.001), or a quadruple asterisk (*p* < 0.0001).

RNA sequencing pathway enrichment analysis identified suppression of the unfolded protein response and endoplasmic reticulum protein folding after induction of *PPP2R3B* (Table S5). Signaling pathway characterization of 302 proteins using reverse phase protein arrays (RPPA, MD Anderson Core) pre- and postinduction of *PPP2R3B* demonstrated enrichment for mammalian target of rapamycin (mTOR) and hypoxia-induced factor 1 (HIF-1) signaling pathways, with prominent biological signatures of response to heat and stress (Fig. 4a–d, Table S6). Immunoblotting provided validation of significantly decreased phosphorylation of AKT at 6–8 hours; however, overall no dramatic effects on known melanoma signaling pathways were demonstrated (Fig. S3a–g). Activation of CDC6, a known direct target of PR70,^17^ was inconsistent across cell lines (Fig. S3f, g).

### The ratio of PR70 to core PP2A enzyme appears to be critical

Increasing molar concentrations of PR70 up to a 1:1 ratio with that of the core PP2A enzyme increased PP2A activity toward its specific substrate pCDC6 in another cellular model; however, further increases in concentration reduced phosphatase activity (Fig. S4). PR70 was shown to be highly efficient at binding to the PP2A core enzyme when competing with another regulatory subunit B’γ1, and overexpression of V5-tagged PR70 in a mammalian cell line (C6) did not lead to free PR70 (Fig. S5), suggesting either competitive binding of other PP2A holoenzymes or direct interactions with other effectors.

### *PPP2R3B* overexpression leads to a significant and sustained rise in expression of previously uncharacterized gene *C21orf91*

**Fig. 5:**
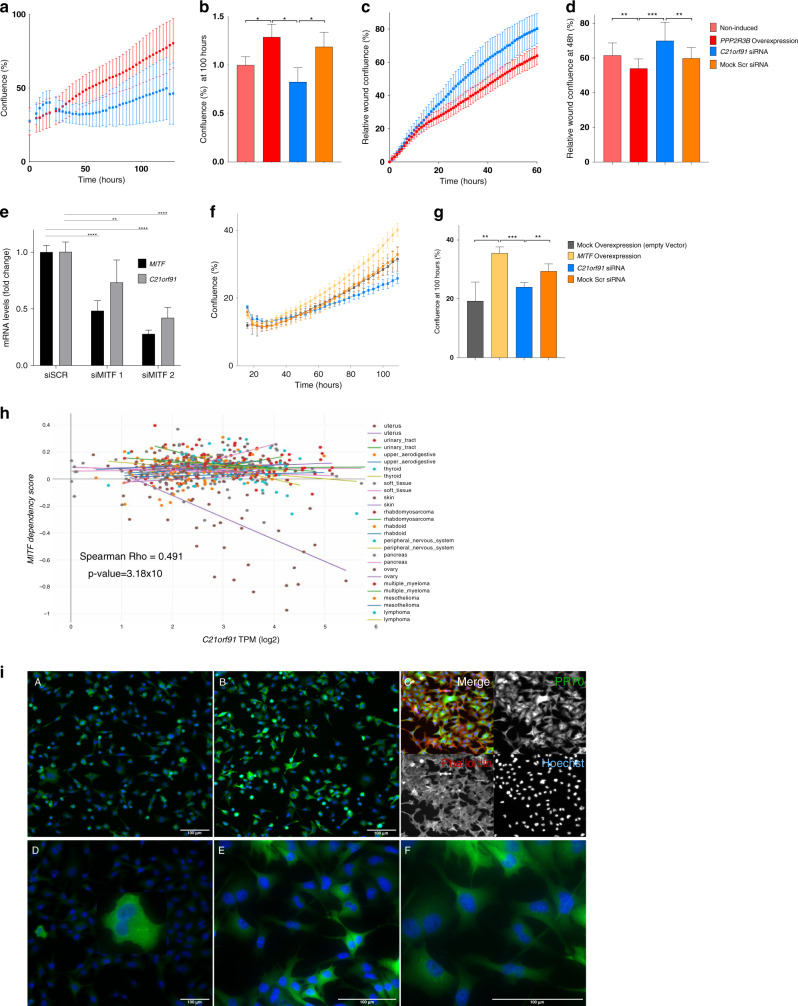
Knockdown of *C21orf91* rescues increased proliferation associated with *PPP2R3B* overexpression. IncuCyte® proliferation assay confluence (%) versus time (hours) for *PPP2R3B*-induced SKMEL2-pTRIPZ-PPP2R3B, with and without small interfering RNA (siRNA) knockdown of *C21orf91* (**a**), with means taken at 100 hours (**b**) (mean confluence at given time point of eight replicates shown with standard deviation) shows a significant increase in proliferation following *PPP2R3B* induction, rescued by *C21orf91* knockdown and significantly different from knockdown with Scr siRNA. Statistical significance was determined using a Student’s *t-*test (Prism v7.0, Graphpad). Statistically significant values are indicated by a single asterisk (*p* < 0.05), a double asterisk (*p* < 0.01), a triple asterisk (*p* < 0.001), or a quadruple asterisk (*p* < 0.0001). Knockdown of *C21orf91* rescues decreased migration associated with *PPP2R3B* overexpression. IncuCyte® scratch wound assay relative wound confluence (%) versus time (hours) for *PPP2R3B*-induced SKMEL2-pTRIPZ-PPP2R3B, with and without siRNA knockdown of *C21orf91* (**c**), with mean taken at 48 hours (**d**) (mean relative wound confluence at each time point of twelve replicates shown with standard deviation) shows a significant decrease in migration following *PPP2R3B* induction, rescued by *C21orf91* knockdown and significantly different from knockdown with Scr siRNA. Statistical significance was determined using a Student’s *t*-test (Prism v7.0, Graphpad). Statistically significant values are indicated by a single asterisk (*p* < 0.05), a double asterisk (*p* < 0.01), a triple asterisk (*p* < 0.001), or a quadruple asterisk (*p* < 0.0001). Knockdown of *MITF* leads to decreased expression of *C21orf91.* Quantitative real-time polymerase chain reaction (qRT-PCR) demonstrating decrease in both *MITF* and *C21orf91* messenger RNA (mRNA) following transfection by two different siRNAs targeting MITF transcript (siMITF 1 and siMITF 2) in SKMEL2 cell line (**e**). Relative fold change in gene expression compared to control cells transfected by nontarget siRNA (siSCRA), standardized to GAPDH, mean + SD of 3 independent experiments. Knockdown of *C21orf91* in cells rescues *MITF-*driven increase in proliferation. IncuCyte® proliferation assay confluence (%) versus time (hours) following overexpression of *MITF* in SKMEL2, with and without siRNA knockdown of *C21orf91* (**f**), with means taken at 100 hours (**g**) (mean confluence at given time point of four replicates shown with standard deviation). *MITF* overexpression drives proliferation as expected, which is rescued by knockdown of *C21orf91*. Controls for both transfection with the MITF overexpression vector and the C21orf91 siRNA are included. Statistical significance was determined using a Student’s *t*-test (Prism v7.0, Graphpad). Statistically significant values are indicated by a single asterisk (*p* < 0.05), a double asterisk (*p* < 0.01), a triple asterisk (*p* < 0.001), or a quadruple asterisk (*p* < 0.0001). Increased expression of *C21orf91* is associated with genetic dependency on MITF in melanoma cell lines. (**h**) CRISPR-Cas9 genome-scale knockout of MITF in melanoma cell lines (*n* = 30) reveals increased expression of *C21orf91* in cells with greater MITF dependency, suggesting that C21orf91 is downstream of MITF. Dependency score as described in https://depmap.org. PR70 and C21orf91 are expressed throughout the cytoplasm with increased expression of C21orf91 in dividing cells. (**i**) Immunocytochemistry of SKMEL30-pTRIPZ-PPP2R3B in uninduced cells at 10× (**A**) and induced cells at 10× (**B**) and 20× (**C**) confirms increased PR70 and C21orf91 expression throughout the cytoplasm following induction of *PPP2R3B*. PR70 is stained with Alex Fluor® 488 (green) secondary antibody, and nuclei stained with Hoescht (blue) and in (**C**) Phalloidin (actin) are visualized with a conjugated Alex Fluor® 647 (far red) antibody. C21orf91 expression is increased in cells with two nuclei, which could be due to various causes including cell division or a cytokinesis defect 10× (**D**), 20× (**E**), 40× (**F**), stained with Alexa Fluor® 488 (green) secondary antibody, and Hoescht nuclear stain (blue). Scale bars represent 100 microns. RE: Inherited duplications of *PPP2R3B* promote nevi and melanoma via a novel *C21orf91*-driven proliferative phenotype.

Given the lack of clear signaling pathway activation in the presence of a pro-proliferative antimigration phenotype, an alternative candidate mediator was sought by unbiased methods. Relatively unknown gene *C21orf91* (Refseq Gene ID:54149) was identified by RNA sequencing as the most significantly differentially expressed gene in both cell lines, and validated at mRNA and protein levels (antibody validated by CRISPR/Cas9 knockout) (Fig. 4e, f, Table S7). Knockdown of *C21orf91* by siRNA rescued both the increased proliferation and decreased migration and measured by Incucyte in SKMEL2 associated with induction of *PPP2R3B* expression (Fig. 5a–d), firmly tying *C21orf91* to the phenotype switch.

### *PPP2R3B*-induced *C21orf91*-driven pigment cell phenotype switching is independent of MITF

Importantly, the *PPP2R3B*-induced increase in *C21orf91* expression was independent of MITF, master regulator of melanocyte transcription and phenotype switching, as witnessed by the lack of MITF overexpression at mRNA and protein levels upon induction of *PPP2R3B* (Fig S2).

### *C21orf91* expression is positively correlated with *MITF* expression in melanoma

Given that MITF is the known master regulator of pro-proliferative phenotype switching in melanoma, and given that the pro-proliferative effect of C21orf91 was not mediated by MITF, we hypothesized that C21orf91 could be an undescribed hub controlling pigment cell phenotype switching, and could therefore be downstream of MITF as well as downstream of PR70. In support, *C21orf91* and *MITF* expression were found to be significantly positively correlated in independent transcriptomic data sets from both melanoma cell lines and the melanoma patient cohort (Fig. S9a–d), implying at least a key role for *C21orf91* in the pro-proliferative state of melanoma, and potentially that *MITF* can operate via *C21orf91*. *MITF* dependency score and *C21orf91* expression were also found to be significantly associated in melanoma cell lines (Fig. 5h), data extracted from the Cancer Dependency Map (Broad Institute).^18–21^ Interestingly, *C21orf91* expression was not positively associated with *PPP2R3B* expression in the same two transcriptomic data sets, with association absent in one and negative in the other.

### C21orf91 expression is regulated by MITF, and mediates MITF-induced proliferation

Knockdown of *MITF* led to decreased expression of *C21orf91* in SKMEL2 cell lines at baseline, demonstrating that *C21orf91* expression can also be regulated by *MITF* (Fig. 5e). Furthermore, increased proliferation in SKMEL2 cells over a five-day Incucyte experiment driven by overexpression of MITF was rescued by knockdown of *C21orf91* (Fig. 5f, g). C21orf91 is therefore a critical molecule controlling proliferation of melanoma cells from at least two pathways, one of which is the canonical pigment-cell phenotype switching pathway driven by MITF.

### PR70 and C21orf91 are expressed throughout the cytoplasm, and their distribution was unchanged by *PPP2R3B* overexpression

PR70 subcellular localization by confocal microscopy was pan-cytoplasmic, including but not restricted to endoplasmic reticulum as previously suggested and not nucleoplasmic as currently suggested,^22^ and increased but unaltered in distribution by overexpression (Fig. 5i). *C21orf91* expression was also demonstrated throughout the cytoplasm, and both PR70 and C21orf91 were increased in cells with two nuclei (Fig. 5i).

## DISCUSSION

Copy number in the genome has in general been less systematically explored than sequence variation due to technical constraints,^23^ as copy-number variation is enriched in areas of low genome mappability.^24^ However, copy-number variants are known to be prevalent in genes for cell communication and RAS-pathway signaling, including serine threonine kinases and phosphatases,^25^ and may therefore be highly relevant in the development of melanoma. Indeed, recent data on rare germline copy-number variants affecting known melanoma susceptibility loci have demonstrated clear proof of concept of copy-number variant predisposition in melanoma families.^26^ Using a rare disease cohort, we identify here new germline duplications in the pseudoautosomal region 1 of the X chromosome that predispose to melanocytic neoplasia. Common to all was *PPP2R3B*, which encodes PR70, ubiquitously expressed in the cytoplasm, and one of the ß” family of regulatory units of the critical phosphatase and regulator of the cell cycle PP2A.^27,28^ PP2A is a heterotrimeric holoenzyme consisting of a structural A subunit, a catalytic C subunit, and a regulatory B subunit.^27,29^ The numerous nonhomologous regulatory subunits are classified into B, B’, B”, and B”’ subfamilies, implicated in control of enzyme activity and substrate specificity.^27,30^ PP2A operates via key effector pathways RAS/MAPK, Wnt, and AKT/mTOR.^27,28^ As such, PP2A activity is intimately involved in malignancy and response to treatments,^31^ and is a major focus of potential therapeutics.^31–34^

Analysis of the TCGA database demonstrates that copy-number variants including *PPP2R3B* are more common across cancers in general than single-nucleotide variants, suggesting that dosage of *PPP2R3B* is relevant in cancer development. In support of these data, a comprehensive study of the role of *PPP2R3B* expression in melanomas at tumor as opposed to germline level found the region to be copy-number sensitive, with loss of the inactivated X in females and decreased expression in males linked to decreased distant metastasis-free survival. The authors proposed that the copy-number sensitivity of this locus could explain the gender differences in melanoma incidence and survival.^35^ It is possible to speculate that despite its location in the pseudoautosomal region 1 (PAR1) of the X chromosome, sex may alter the effects of *PPP2R3B* expression in the germline as well. As our patients with duplications were however of both sexes, and the correlation between *PPP2R3B* expression and survival in the melanoma trancriptomic data was independent of sex, we do not currently have any evidence for such an effect.

Having discovered germline duplications in gene *PPP2R3B* in cohorts of individuals with melanocytic neoplasia, we sought to understand the mechanism of action. Our data demonstrate that *PPP2R3B* overexpression promotes proliferation of *NRAS*-mutant melanoma cell lines, which could explain the predisposition to the development of a clinically apparent melanocytic nevus or melanoma in the context of a somatic pathogenic variant in a melanocyte. Alternatively, the pro-proliferative germline environment could in and of itself predispose to somatic pathogenic variant in the skin, via increased cell division or alteration of cell cycle regulation and the associated effects on DNA repair. As we do not observe deletions in patient cohorts, only duplications, siRNA knockdown was not expected to produce biological effects. None of the CMN patients with duplications have so far developed melanoma; however, this is in line with what would be expected statistically, as the incidence of melanoma in CMN at this age is very low,^36^ and no conclusions can yet be drawn about potential association between the *PPP2R3B* duplications and outcome in CMN. Interestingly, however, our data demonstrate clearly that increased *PPP2R3B* expression correlates with improved disease-specific survival in melanoma, mirroring the significant protective effect of *PPP2R3B* expression in urothelial cancer and pancreatic cancer data sets from the TCGA database.^37^ A significant association between expression and survival is not seen in the smaller melanoma TCGA data set; however, this may be due to lack of statistical correction for known associated factors. In the larger melanoma cohort studied here, improved survival appears to be mediated via a nonimmunological mechanism, and could potentially operate via phenotype switching toward proliferation and away from migratory potential (i.e., less metastatic potential).

Pigment cell phenotype switching is classically controlled by a reciprocal relationship between *MITF* and *POU3F2*;^7,8,38,39^ however, we demonstrate clearly here that *PPP2R3B*-induced pigment cell phenotype switching is MITF-independent, and is instead driven by the relatively uncharacterized gene *C21orf91*. Although this mechanism could have involved the AKT/mTOR/pS6K pathway, signaling pathway alterations were largely unimpressive as measured by unbiased RPPA and by candidate immunoblotting, and indeed protein modeling demonstrated a decrease in phosphatase activity with an increasing ratio of PR70 to the core enzyme.

We therefore hypothesized that if *PPP2R3B*-induced pigment cell phenotype switching was operating via *C21orf91*, perhaps MITF-induced proliferation also operates via *C21orf91*. This hypothesis is supported by the significant association between *MITF* and *C21orf91* expression in a melanoma cohort and pooled melanoma cell lines. Further supportive evidence for a role of *C21orf91* in this field includes its previous identification within a pro-proliferative anti-invasive transcriptomic signature in melanoma,^40,41^ a central role in cell phenotype determination in neurological development,^42^ and recognition as one of 180 key molecules in cross-species protein networks around the Ras-MAPK/PI3K pathways.^43^ *MITF* knockdown in melanoma cells did indeed suppress *C21orf91* expression at baseline, and in a five-day real time proliferation assay *MITF* overexpression driving melanoma cell proliferation was rescued by knockdown of *C21orf91*.

The lack of positive association between *C21orf91* expression and *PPP2R3B* expression in the two transcriptomic data sets is potentially due to the multiple inputs to *C21orf91* as a hub, but could also be influenced by copy-number changes to Xp that are relatively common in melanoma, affecting *PPP2R3B* expression.

Due to the highly repetitive nature of this region of the genome near the telomeric end of Xp, we found targeted NGS to be the most reliable way to detect and confirm duplications of *PPP2R3B*. Multiple custom-designed TaqMan® copy-number assays (Thermo Fisher Scientific, USA) were insufficiently robust for diagnostic validation. Future screening of larger melanoma cohorts and families will likely require development of a diagnostic-grade test from the point of view of cost, which will allow assessment of the frequency across different cohorts, of the penetrance of the melanoma phenotype associated with this variant, and association with clinical outcome.

We identify here germline duplications in the gene *PPP2R3B* predisposing to nevogenesis and melanoma in an important proportion of cases. Duplications increase melanocytic tissue expression of the protein product PR70, which confers a survival advantage in the context of melanoma, possibly via promotion of a pro-proliferative and antimigratory pigment cell phenotype. This phenotype in vitro is driven by an undescribed MITF-independent mechanism mediated by *C21orf91*. This work offers novel insights into both the origins and behavior of melanocytic neoplasia, and identifies *C21orf91* as an important new and potentially targetable fulcrum in the control of proliferation.

## Supplementary information

Supplementary materials and methods

Supplementary information

Supplementary table 3

Supplementary table 4

Supplementary table 5

## Data Availability

Array CGH data have been submitted to http://www.ncbi.nlm.nih.gov/clinvar/. Melanoma transcriptomic data were deposited into the European Genome-phenome Archive (EGA) (accession no. EGAS00001002922 - https://ega-archive.org/studies/EGAS00001002922), access request to j.a.newton-bishop@leeds.ac.uk. Cell line *PPP2R3B* overexpression RNAseq data were deposited at the Gene Expression Omnibus (GEO) with accession number GSE145195, https://www.ncbi.nlm.nih.gov/geo/query/acc.cgi?acc=GSE145195, access request to veronica.kinsler@crick.ac.uk.
