# Supplementary material for: Inherited duplications of PPP2R3B predispose to nevi and melanoma via a C21orf91-driven proliferative phenotype
Source: Genet Med. 2021 Jun 18;23(9):1636–47. doi: 10.1038/s41436-021-01204-y (PMC8460442; doi:10.1038/s41436-021-01204-y)
Supplement: Supplementary file 1 — Supplementary materials and methods [file 41436_2021_1204_MOESM1_ESM.pdf]

Supplementary Materials

Materials and Methods

Patient recruitment and approvals

All participants gave written informed consent as part of ethically approved studies. CMN patients were recruited from Great Ormond Street Hospital (GOSH) London, with London Bloomsbury Research Ethics Committee (REC) approval, and melanoma samples were obtained from Professor Newton-Bishop, Leeds, with approval from North East – York UK REC. DNA was extracted from blood samples using standard methods in all cohorts.

Sample preparation for Reverse Phase Protein Array and RNAseq

SKMEL30 pTRIPZ-PPP2R3B and SKMEL2 pTRIPZ-PPP2R3B cells were seeded and induced with doxycycline 1µg/ml for either 6 hours or 16 hours alongside untreated controls. Samples were collected in triplicate for both RNA extraction and protein lysis. Confirmation of *PPP2R3B* induction was performed by qPCR and Western blotting.

Immunohistochemistry

PR70 expression was assessed using an optimised protocol with antibody against PR70 (Key Resources - Table S1). FFPE sections were dewaxed, rehydrated and heat mediated antigen retrieval was performed. Sections were blocked for one hour at room temperature in 1% FBS 10% BSA prior to incubation in primary antibody in blocking solution at 1:50 overnight at 4°C. Sections were incubated with HRP-tagged secondary antibody at 1:500 for one hour at room temperature. Samples were treated with hydrogen peroxide 0.3% for 10 minutes at room temperature prior to application of DAB chromogen for 6 minutes, following which sections were dehydrated and mounted. Between steps sections were washed three times for 5 minutes in PBS Tween 0.05%. Slides were scanned using a Zeiss Axio Scan.X1 slidescanner at 20X. Staining was graded in naevus nests as 0-3 intensity of DAB staining by two blinded assessors (SP, VK).

Quantitative Real Time PCR

1µg of total RNA was reverse transcribed and quantitative real time PCR performed, using Taqman probes (Key Resources - Table 2) and the StepOnePlus Real-Time PCR System (ABI, Abilene, TX, USA). Relative fold change to the housekeeping gene *GAPDH* was calculated by the ΔΔCT method.

Western Blotting

Protein lysate was quantified by Bradford protein assay, and 20ug of each sample run on a 10% Bis-Tris protein gel. PVDF membrane was activated in methanol prior to wet transfer. The membrane was washed three times in TBST, then blocked using 3% albumin at room temperature for one hour. Membranes were then incubated in primary antibody (**Table S1**) in 3% albumin overnight at 4°C. Membranes were washed three times in TBST then incubated in corresponding HRP-tagged secondary antibody in 5% milk for 1 hour at room temperature. Membranes were washed a further three times in TBST before adding ECL for two minutes followed by measurement of chemiluminescence using Amersham Imager 600. Intensity of bands was quantified using ImageJ software.

Immunocytochemistry

4 x 10^4 cells were seeded per well in laminin-coated 4-well chamber slides (Millicell® EZ slide, Merck Millipore, PEZGS0816), washed in cold PBS and fixed using 4% paraformaldehyde (PFA), followed by permeabilisation using 0.1% Triton X100. After three washes with PBS they were incubated with blocking solution (1% Bovine serum albumin, 10% Donkey serum, 0.3M Glycine, 0.1% PBS Tween) for 1 hour at room temperature followed by primary antibody (**Table S1**) in blocking solution overnight at 4°C. Slides were washed once with 1:10,000 Hoechst, three times with 0.1% PBS Tween, and incubated with appropriate fluorescent secondaries at room temperature for one hour in the dark. Slides were again washed three times with 0.1% PBS Tween and mounted prior to analysis.

MLPA®

Customised MLPA® was performed as per MRC-Holland MLPA® DNA protocol version MDP-v003, optimized using 25ng of DNA per well, and a 1 in 2 dilution of the customised probemix, using four probes for *PPP2R3B,* and one each for *GTPBP6* and *PLCXD1* designed to dovetail by

size with the pan-genome probes of known normal copy number in the reference SALSA® MLPA® probemix P200-A1 (MRC-Holland). Capillary electrophoresis of diluted PCR products with ROX-500 size standard was performed on an ABI 3730xl, and data analysed using GeneMarker® v2.4 (SoftGenetics, USA). Each plate contained the same controls of normal copy number and duplication for standardisation. A duplication was determined if all four probes within PPP were at a ratio of 1.3:1 or higher, whilst all the control probes were at 1:1 ratio.

Validation of antibodies by CRISPR/Cas9 knockout

Antibodies for PR70 (*PPP2R3B* - Abcam ab234731) and C21orf91 (Sigma HPA049030 and Abcam ab80695) were validated using CRISPR/Cas9 knockout of the genes in question in the HEK293 cell line. CRISPR/Cas9 knockouts were generated using the Alt-R CRISP-Cas9 system (Integrated DNA Technologies). Briefly, three guide RNAs per gene were designed and produced as crRNAs (see Key resources - Table 3). Duplex guide RNAs were produced following incubation of each crRNA with a fluorescently labelled tracerRNA at 95°C, which were then complexed with Cas9 protein to form a ribonucleoprotein (RNP) transfection complex. Reverse transfection of three different RNP complexes plus a pooled RNP complex for each of the two genes was achieved using CRISPRMAX™ (Thermofisher, Invitrogen), and addition of 50µl of RNP complex (final concentration of both guide RNA and Cas9 60nM) to 8 x 10^5 HEK cells in one well of a 6 well plate. Cells were incubated at 37°C, 5% CO_2 for 24 hours prior to single cell sorting by flow cytometry of the population of cells containing the fluorescent tracerRNA into a 96 well plate. Single cell colonies were expanded to 6 well plates and DNA extracted using QuickExtract DNA extraction solution (Lucigen) and protein lysate harvested by standard methods. Sanger sequencing confirming frameshift indels, and immunoblotting for each protein product in the presence of control cell lines confirmed complete protein absence **(Figure S5)** and therefore target specificity of each antibody.

***PPP2R3B* knockdown IncuCyte® Cell Count Proliferation Assay**

SKMEL2 cells were seeded into a 96 well ImageLock plate at a density of 1 x 10^4 cells per well. *PPP2R3B* knockdown was achieved in selected cells by transefction with Lipofectamine™ RNAiMAX using a pooled *PPP2R3B* siRNA (ON-TARGETplus Human *PPP2R3B*, Dharmacon 28227) at 10nM alongside mock and scrambled siRNA transfections. Efficacy of knockdown

at mRNA level was confirmed at concentrations of 10nM and 25nM by qRT-PCR (Figure S6a). The plate was then analysed using the IncuCyte® live-cell analysis system and set up to acquire 10X phase contrast images at a scanning interval of 60 minutes for 5 days, measuring percentage confluence. Confluence was averaged between 12 replicates for each condition (mean + SD) and statistical significance was calculated by Students t-test **(Figure S6b-d)**.

Protein preparation, and holoenzyme assembly with PR70 and B'γ1 regulatory subunits via GST-mediated pull-down assay

Expression and purification of PP2A Aα (9-589), Cα (1-309), PR70 subunit, and Cdc6, and assembly of the PP2A core enzyme (Aα-Cα heterodimer) followed procedures described previously. Approximately 10 µg of GST-AC (core enzyme) was bound to 10µl of glutathione resin via GST tag. The resin was washed with 200µl assay buffer three times to remove the excess unbound protein. Then, the indicated amounts of PR70 and B'γ1 were added to the resin in a 200µl volume suspended in the assay buffer containing 25 mM Tris (pH 8.0), 150 mM NaCl, 1 mM $CaCl_2$, and 3 mM DTT. The mixture was washed three times with the assay buffer. The proteins remained bound to resin were examined by SDS-PAGE, and visualized by Coomassie blue staining. All experiments were repeated three times. The level of binding was quantified using Image J, and results from three separate experiments were fitted in GraphPad Prism (GraphPad Software, Inc.).

Phosphatase assay

Purified GST-Cdc6 (49-90) was phosphorylated *in vitro* by Cyclin A/CDK2 (1/20 w/w) with 10 mM $MgCl_2$ and 10x molar concentration of ATP for 1 hour at 30°C. The phosphorylated protein was purified by gel filtration chromatography (Superdex 200, GE Healthcare) to remove free ATP, followed by overnight cleavage of the GST-tag with TEV protease (1/20 w/w). The pCdc6 peptide was then separated from GST or uncleaved peptide using untrafiltration membrane (Millipore) with a 10 kDa cut-off. The phosphatase assay was performed in 100µl assay volume (rather than 50 µL) at room temperature for 15 minutes and stopped by the addition of 2x malachite green (50 µL) rather than 100 µL of 1x malachite green to increase the sensitivity of the assay. The phosphatase activity of 0.16 µM of PP2A

core enzyme was measured using 2.2 µM pCdc6 peptide in a buffer containing 25 mM Tris pH 8.0, 150 mM NaCl, 3mM DTT, 50 µM $MnCl_2$ and 1 mM $CaCl_2$, in the presence and absence of the indicated concentrations of PR70 (108-519). The absorbance at 620 nm was measured after 10 min incubation at room temperature. The PR70 concentration-dependent PP2A phosphatase activity toward pCdc6 was fitted in GraphPad Prism (GraphPad Software, Inc.) by two separate simulations for two ranges of PR70 concentrations, lower versus higher than the molar concentration of PP2A core enzyme. The experiments were repeated three times, representative results were shown.

Mammalian cell culture, recombinant expression of PR70, and fractionation of cell lysate via gel filtration chromatography followed by western blot

293T and C6 glioma cells were cultured in Dulbecco's modified Eagle's medium (DMEM) supplemented with 10% fetal bovine serum (FBS), 100 units/ml of penicillin, and 100 µg/ml of streptomycin. The V5-tagged human PR70 (V5-PR70) was cloned into murine leukemia retroviral vectors harboring a CMV promoter for over-expression. Retroviruses harboring V5-PR70 were packaged in 293T cells, and were used to infect 293T or C6 glioma cells with 50-80% confluence for over-expression of V5-PR70. The infection efficiency of retroviruses was monitored by the fluorescence signals of RFP or GFP included in retroviral vectors. The level of recombinant V5-PR70 in holoenzyme versus as free subunit were determined by fractionation of cell lysate over gel filtration chromatography to examine co-migration of V5-PR70 with the scaffold A and PP2Ac subunits. The relative amounts of A, PP2Ac, and V5-PR70 in each fraction were determined by western blot using antibodies that specifically recognize A (Millipore), PP2A Cα subunit (Millipore, 1D6), and V5-tag (Millipore), followed by quantification of the western blot signals using Image J. The experiment was repeated three times; representative results are shown.

Key Resources

Supplemental Table 1 - Antibodies:

Antibody	Source	Catalogue Number	Dilution
**PPP2R3B**	Rabbit	Abcam, ab72027	1:1000 Western Blot<br>1:500 Immunocytochemistry
**MITF**	Mouse	Abcam ab3201	1:1000
**Vinculin**	Mouse	Cell signaling technology #4650S	1:1000
**C21orf91**	Rabbit	Altas Antibodies, HPA018288	1:1000 Western Blot<br>1:500 Immunocytochemistry
**MYC-tag**	Mouse	Abcam ab32	1:2000 Western Blot<br>1:500 Immunocytochemistry
**S6K1**	Rabbit	Abcam ab32529	1:1000
**AKT**	Rabbit	Cell signaling technology #4685	1:1000
**Phospho AKT S473**	Rabbit	Cell signaling technology #9271	1:1000
**Phospho AKT T308**	Rabbit	Cell signaling technology #13038	1:1000
**MYC**	Mouse	Abcam ab32072	1:1000

Supplemental Table 2 - Taqman® probes:

TaqMan® Probe 20X	Source, Catalogue Number
PPP2R3B	Hs00203045_m1
C21orf91	Hs00213743_m1
GAPDH	Hs02786624_g1
MITF	Hs01117294_m1

Figure S1.

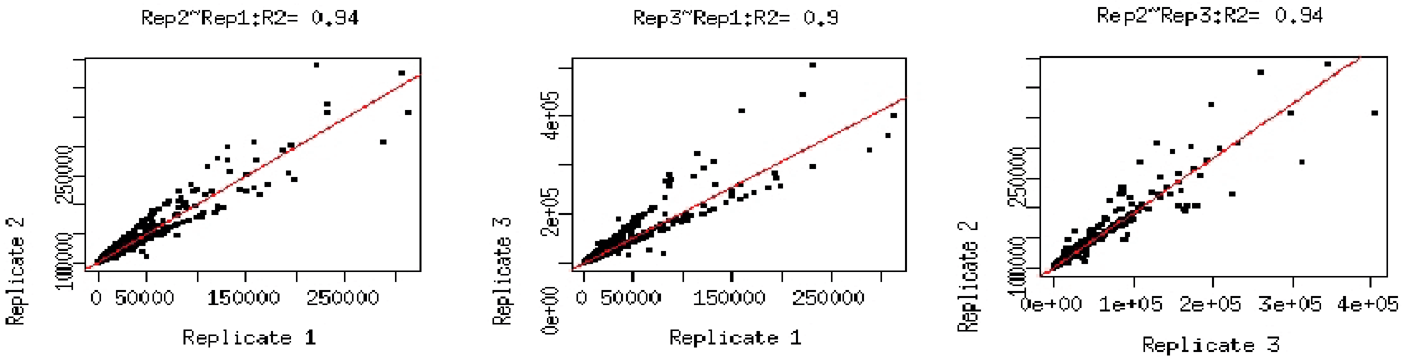

Figure S2.

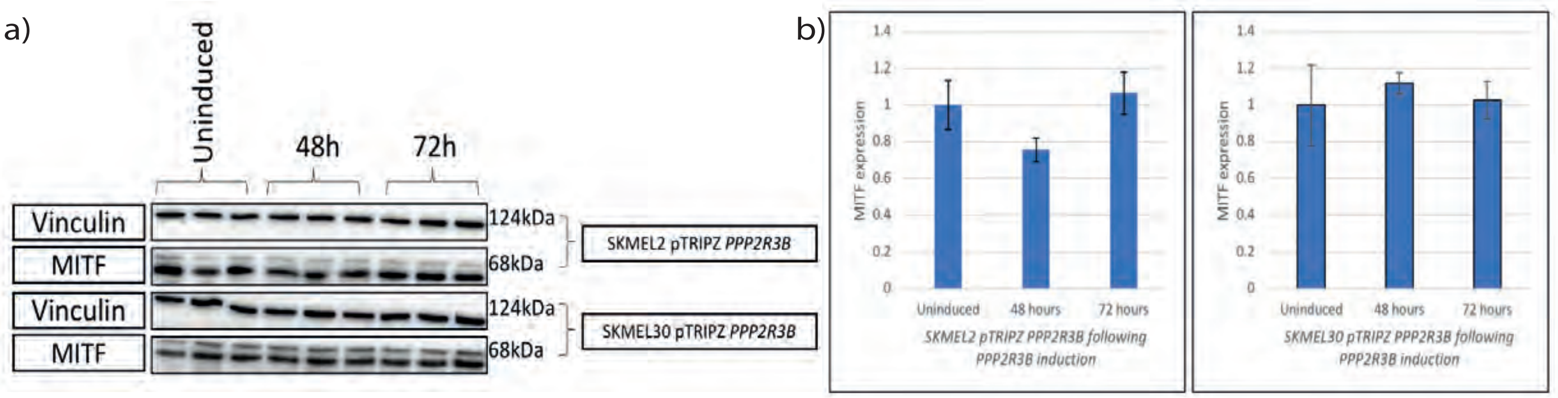

Figure S1

Replicates of induced and uninduced cells used in RNAseq show good correlation. From left to right, correlation between 1st and 2nd replicates, 1st and 3rd replicates and 2nd and replicates.

Figure S2

Overexpression of *PPP2R3B* does not induce proliferation via MITF. Western blot of uninduced and induced SKMEL2 pTRIPZ *PPP2R3B* and SKMEL30 pTRIPZ *PPP2R3B* cells for MITF expression **(a),** and quantification **(b),** show *PPP2R3B* overexpression does not consistently alter MITF activation across the cell lines in this system, suggesting the increased proliferation in cell lines observed following *PPP2R3B* overexpression is MITF independent

Figure S3.

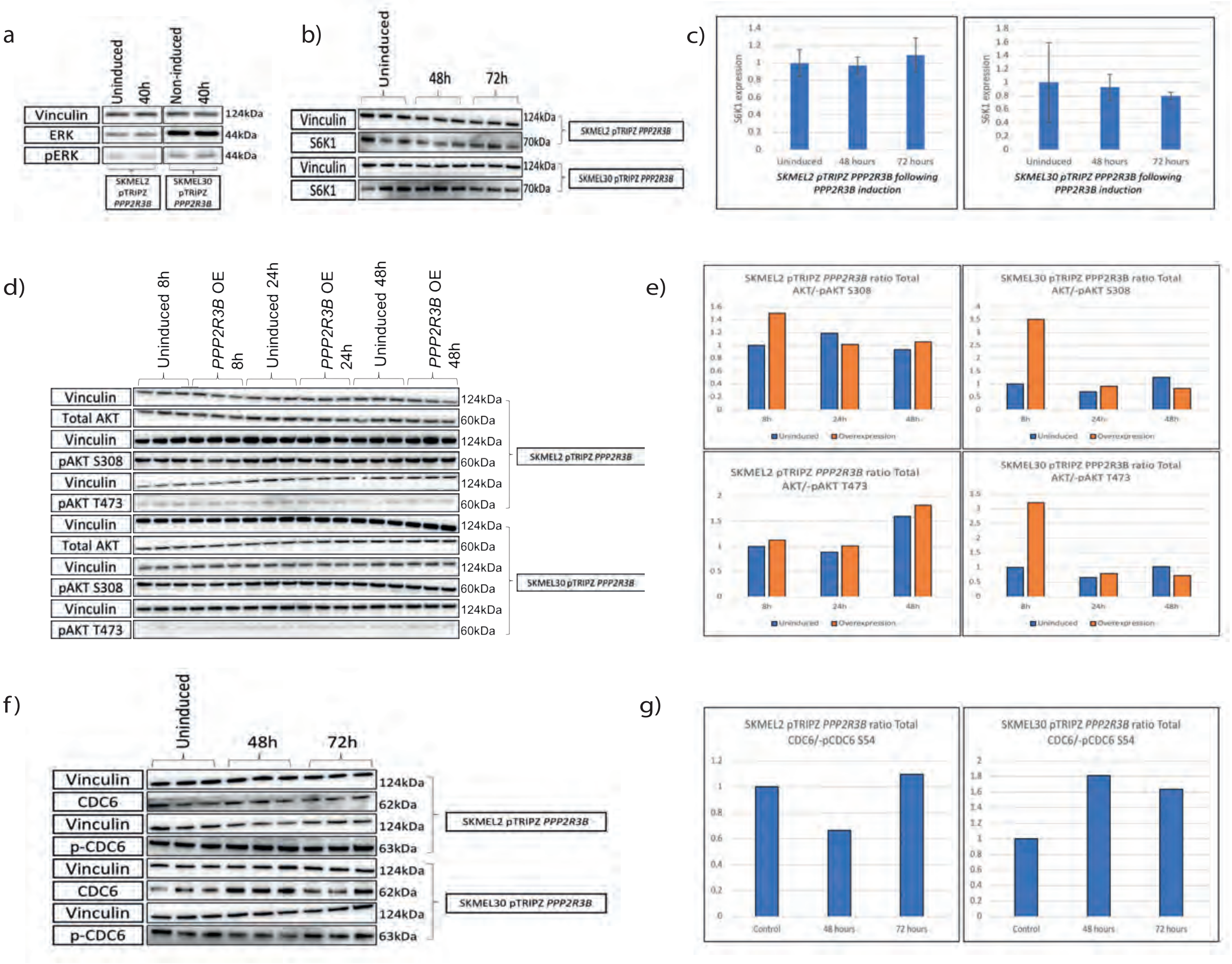

a
Uninduced
40h
Non-induced
40h
Vinculin
ERK
pERK
124kDa
44kDa
44kDa
SKMEL2 pTRIPZ PPP2R3B
SKMEL30 pTRIPZ PPP2R3B
b)
Uninduced
48h
72h
Vinculin
S6K1
124kDa
70kDa
SKMEL2 pTRIPZ PPP2R3B
SKMEL30 pTRIPZ PPP2R3B
c)
S6K1 expression
Uninduced
48 hours
72 hours
SKMEL2 pTRIPZ PPP2R3B following PPP2R3B induction
SKMEL30 pTRIPZ PPP2R3B following PPP2R3B induction
d)
Uninduced 8h
PPP2R3B OE 8h
Uninduced 24h
PPP2R3B OE 24h
Uninduced 48h
PPP2R3B OE 48h
Vinculin
Total AKT
pAKT S308
pAKT T473
124kDa
60kDa
SKMEL2 pTRIPZ PPP2R3B
SKMEL30 pTRIPZ PPP2R3B
e)
SKMEL2 pTRIPZ PPP2R3B ratio Total AKT/-pAKT S308
SKMEL30 pTRIPZ PPP2R3B ratio Total AKT/-pAKT S308
SKMEL2 pTRIPZ PPP2R3B ratio Total AKT/-pAKT T473
SKMEL30 pTRIPZ PPP2R3B ratio Total AKT/-pAKT T473
8h
24h
48h
Uninduced
Overexpression
f)
Uninduced
48h
72h
Vinculin
CDC6
p-CDC6
124kDa
62kDa
63kDa
SKMEL2 pTRIPZ PPP2R3B
SKMEL30 pTRIPZ PPP2R3B
g)
SKMEL2 pTRIPZ PPP2R3B ratio Total CDC6/-pCDC6 S54
SKMEL30 pTRIPZ PPP2R3B ratio Total CDC6/-pCDC6 S54
Control
48 hours
72 hours

Figure S3

Overexpression of *PPP2R3B* dephosphorylates AKT, but does not consistently affect CDC6 activation.

Western blots of lysate from uninduced and induced SKMEL2-pTRIPZ-PPP2R3B and SKMEL30-pTRIPZ-PPP2R3B cells, for (a) ERK and phospho-ERK, (b,c) S6K1 with quantification, (d,e) AKT and phospho-AKT (S308 and T473) with quantification of ratio of total to phospho-AKT, demonstrate *PPP2R3B* overexpression decreases phosphorylation of AKT at 6-8 hours, but does not lead to strong activation of known melanoma signalling pathways. Western blots of (f,g) CDC6 and phospo-CDC6 and quantification of ratio of total to phospho-CDC6, show *PPP2R3B* overexpression does not consistently alter CDC6 activation across the cell lines in this system. This is supported by direct phosphatase activity measurements in another cellular model demonstrating that the interaction between PR70 and CDC6 may be dose-dependent (Figure S4).

Figure S4.

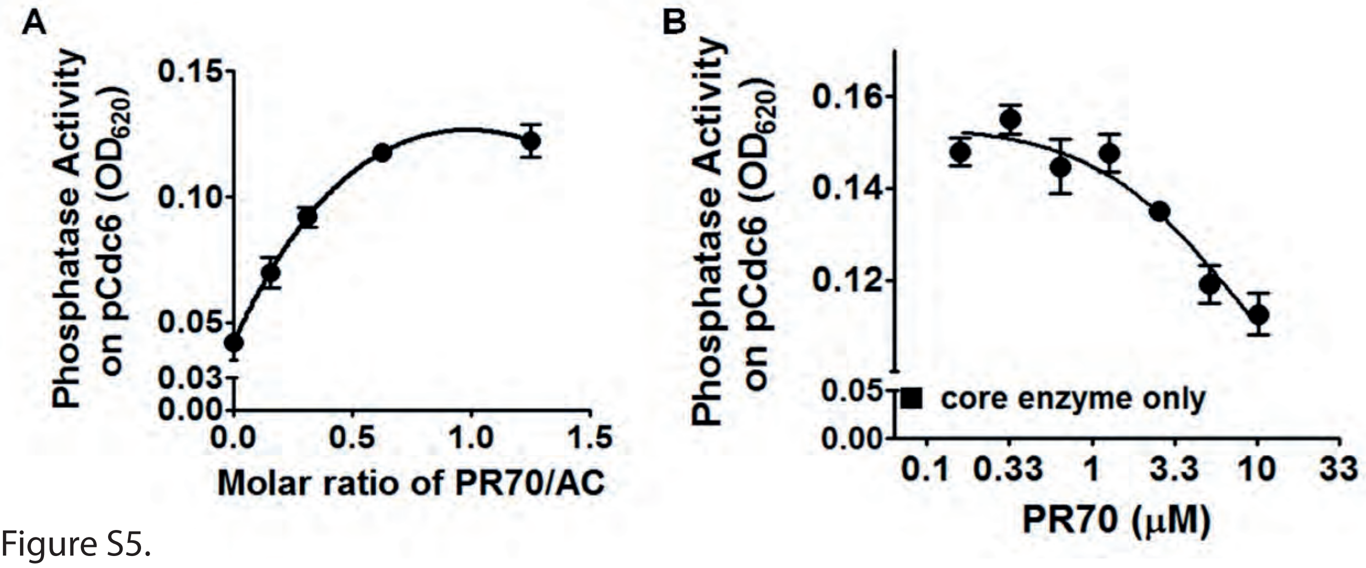

Figure S5.

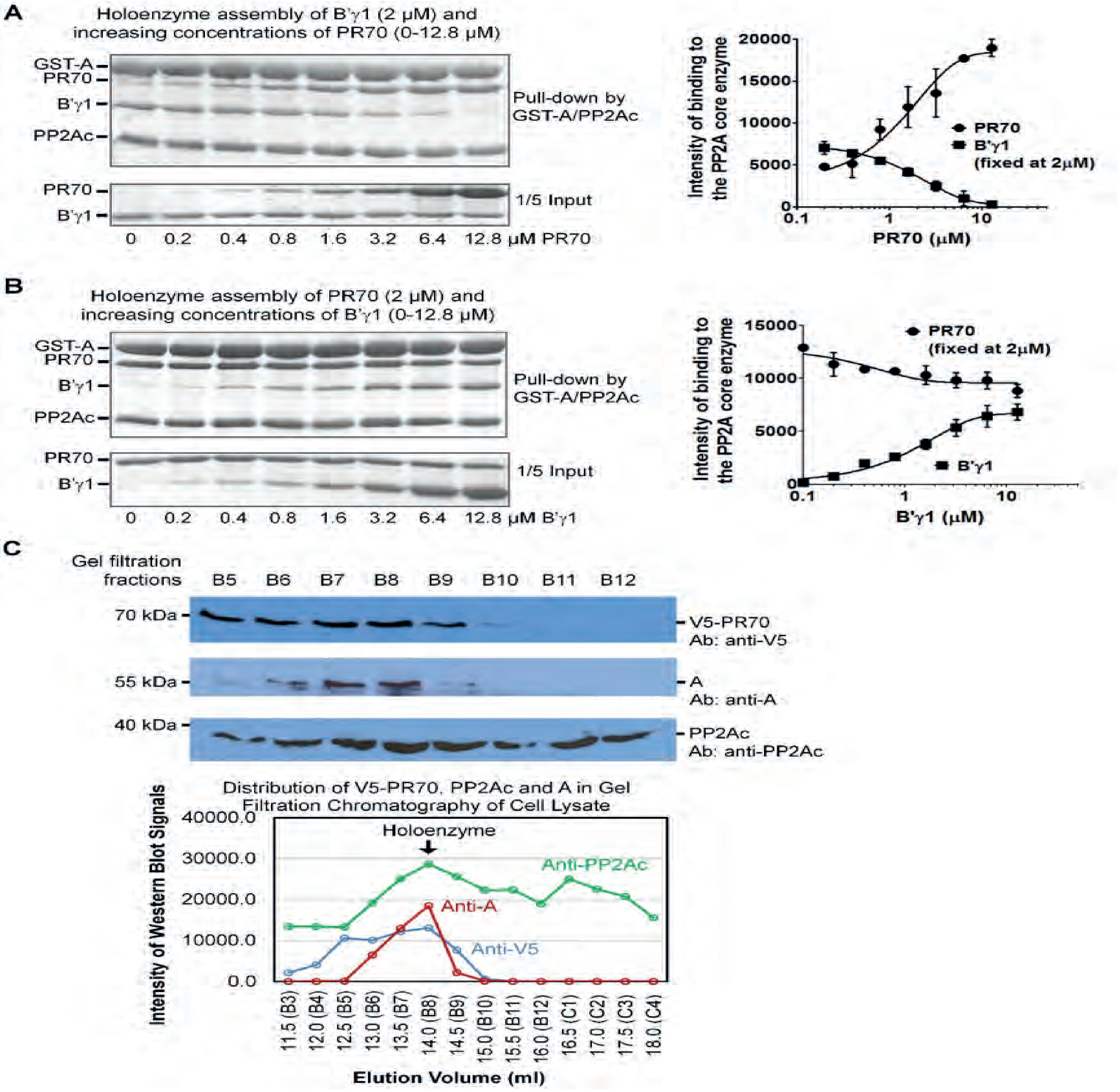

Figure S4

Increasing concentrations of PR70 gave increased PP2A activity toward its specific substrate, pCdc6, a cell cycle regulator that participates in tight control of replication licensing[41] **(a)**. However, when the molar concentration of PR70 was higher than PP2A core enzyme, further increase of PR70 level reduced the phosphatase activity of PP2A, possibly due to a dominant negative effect via competitive inhibition **(b)**.

Figure S5

PR70 efficiently competes with the B'γ1 regulatory subunit for holoenzyme assembly

A). Pull-down of a fixed concentration of B'γ1 in the presence and absence of increasing concentrations of PR70 via GST-tagged PP2A core enzyme (A/PP2Ac). The bound proteins were visualized on SDS-PAGE by Coomassie blue staining. PR70 efficiently reduced the holoenzyme assembly with the B'γ1 regulatory subunit. B). Pull-down of a fixed concentration of PR70 in the presence and absence of increasing concentrations of B'γ1 similar to that described in A). B'γ1 could barely compete with PR70 for holoenzyme assembly. For both panel A) and B), the experiments were repeated three times; representative results are shown. The simulation of the quantified data from three repeats was shown on the right. C). Western blot detection of A, PP2Ac, and recombinant V5-PR70 in fractions of gel filtration chromatography of cell lysates with overexpressed V5-PR70. The distribution curves of the three components are shown in the lower panel.

Figure S6.

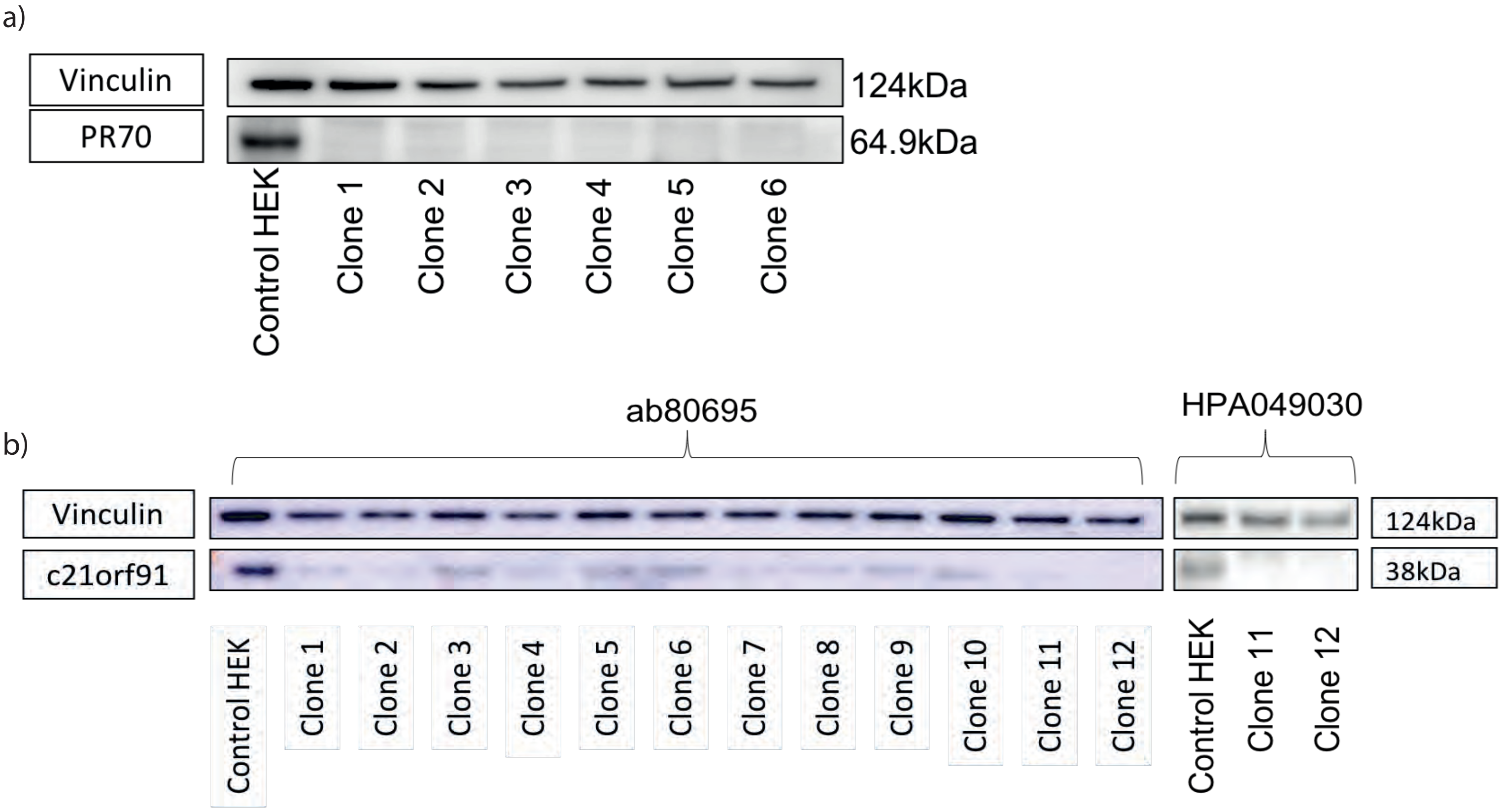
a)
Vinculin
PR70
124kDa
64.9kDa
Control HEK
Clone 1
Clone 2
Clone 3
Clone 4
Clone 5
Clone 6
b)
ab80695
HPA049030
Vinculin
c21orf91
124kDa
38kDa
Control HEK
Clone 1
Clone 2
Clone 3
Clone 4
Clone 5
Clone 6
Clone 7
Clone 8
Clone 9
Clone 10
Clone 11
Clone 12
Control HEK
Clone 11
Clone 12

Figure S6

Validation of PR70 antibody (Abcam ab234731) and C21orf91 antibodies (Sigma, HPA049030 and Abcam ab80695) using CRISPR-Cas9 knockout cell lines

Western blot of a) PPP2R3B CRISPR-Cas9 HEK293 single cell clones demonstrates no expression of PR70 in comparison to control protein lysate from HEK293 and b) C21orf91 CRISPR-Cas9 HEK293 single cell clones demonstrates no expression of C21orf91 in knockout clones 11 and 12 using both C21orf91 antibodies in comparison to control protein lysate from HEK293. Corresponding Sanger sequencing from knockout cell lines confirms knockout.

Figure S7.

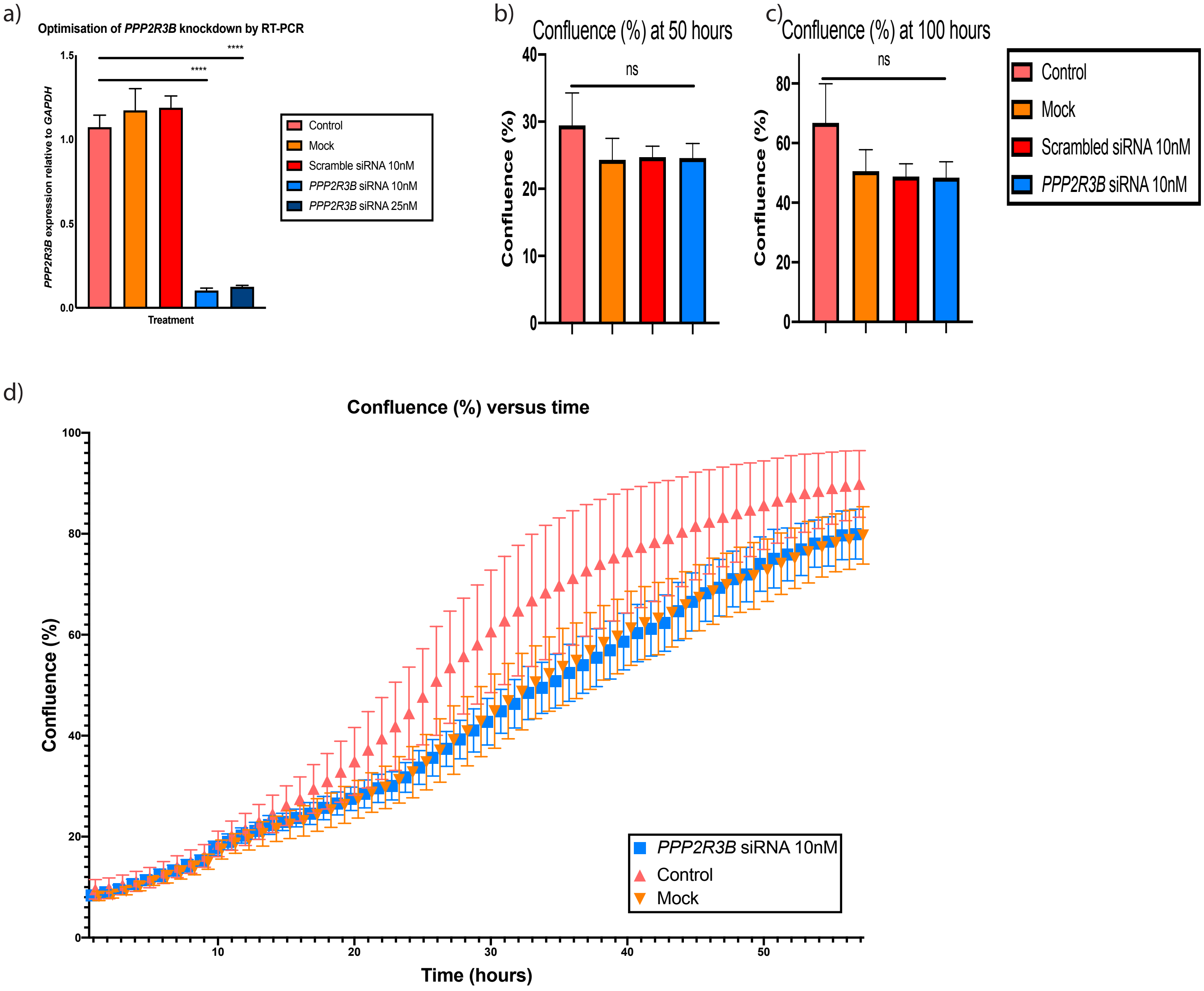
a)
Optimisation of PPP2R3B knockdown by RT-PCR
PPP2R3B expression relative to GAPDH
Treatment
****
****
Control
Mock
Scramble siRNA 10nM
PPP2R3B siRNA 10nM
PPP2R3B siRNA 25nM
b)
Confluence (%) at 50 hours
Confluence (%)
ns
c)
Confluence (%) at 100 hours
Confluence (%)
ns
Control
Mock
Scrambled siRNA 10nM
PPP2R3B siRNA 10nM
d)
Confluence (%) versus time
Confluence (%)
Time (hours)
PPP2R3B siRNA 10nM
Control
Mock

Figure S7

***PPP2R3B* knockdown is not shown to have a significant effect on proliferation in SKMEL2**

Validation of PPP2R3B knockdown in SKMEL2 demonstrates efficient knockdown of PPP2R3B at RNA level at siRNA concentrations of 10nM and 25nM in comparison to control, mock and scrambled siRNA 10nM by RT-PCR **(a).** Mean expression of four replicates is shown with standard deviation. Statistical significance was determined using a Student's t-tests. No significant effect on proliferation is observed following *PPP2R3B* knockdown in SKMEL2 using IncuCyte® cell count proliferation assay over 100 hours, measuring confluence (%) versus time (hours) in comparison to control, mock and scrambled siRNA 10nM **(b)** (mean confluence at each time point of eight replicates shown with standard deviation). The mean confluence with standard deviation is shown at timepoints of 50h **(c)** and 100h **(d).** Statistical significance was determined using a Student's t-tests at each time point.

Figure S8.

a)

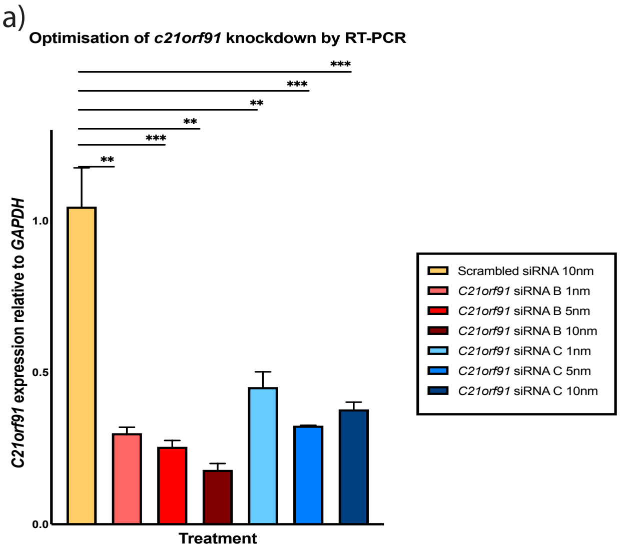

b)

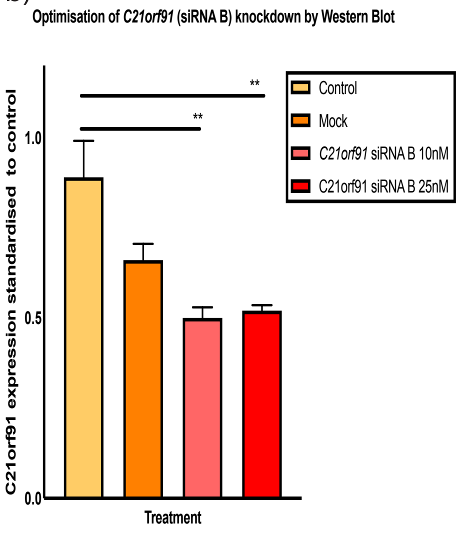

c)

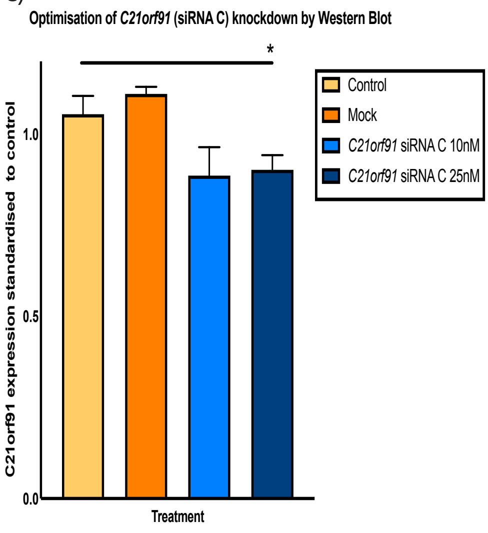

Figure S8

Validation of *C21orf91* knockdown in SKMEL2

Validation of *C21orf91* knockdown in SKMEL2 demonstrates efficient knockdown of two *C21orf91* siRNAs at RNA level at concentrations of 1nM, 5nM and 10nM in comparison to scrambled siRNA 10nM by RT-PCR **(a).** Mean expression of four replicates is shown with standard deviation. Statistical significance was determined using a Student's t-tests. C21orf91 knockdown demonstrated at protein level by Western blot using two *C21orf91* siRNAs 10nM in comparison to control and mock transfection, siRNA B **(b)** and siRNA c **(c)**. Mean expression of three replicates is shown with standard deviation. Statistical significance was determined using a Student's t-tests.

Figure S9.

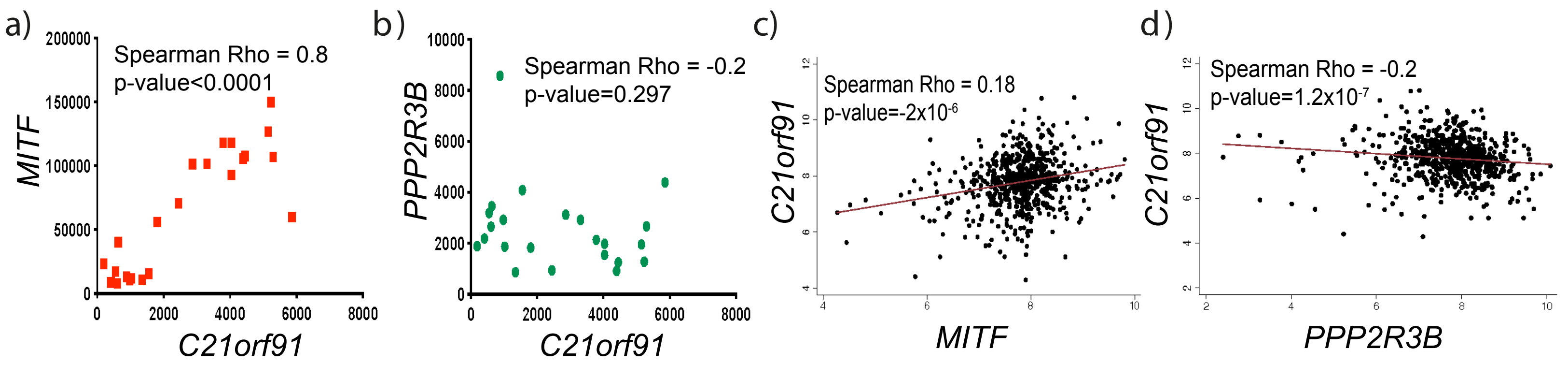
a)
Spearman Rho = 0.8
p-value<0.0001
MITF
C21orf91
b)
Spearman Rho = -0.2
p-value=0.297
PPP2R3B
C21orf91
c)
Spearman Rho = 0.18
p-value=-2x10-6
C21orf91
MITF
d)
Spearman Rho = -0.2
p-value=1.2x10-7
C21orf91
PPP2R3B

Figure S9

C21orf91 and MITF expression are significantly positively correlated in melanoma, independent of PPP2R3B.

(a) Positive correlation observed between expression of between expression of *C21orf91* and *MITF* demonstrated in independent transcriptomic data from a cohort of 24 human derived melanoma cell lines, Spearman Rho 0.8 (95% CI 0.6-0.9, p<0.0001), but (b) no correlation observed between expression of *C21orf91* and *PPP2R3B*. Similarly, (c) positive correlation demonstrated in independent transcriptomic data in human melanoma tissue Spearman Rho 0.18 ($p = 2 \times 10^{-6}$) but (d) negative correlation observed between expression of *C21orf91* and *PPP2R3B,* Spearman Rho -0.2 ($p = 1.2 \times 10^{-7}$). This points to increased expression of *C21orf91* as a new hub in melanocytic proliferation, not only a target of *PPP2R3B*. Increased expression of *C21orf91* is associated with genetic dependency on MITF in melanoma cell lines.

Table S3 – Upregulated genes associated with *PPP2R3B* expression are enriched for non-immune pathway signatures - transcriptomic data from 703 melanoma tumour samples from Leeds melanoma cohort (see methods for details)

Table S4 - Down regulated genes associated with *PPP2R3B* expression are enriched for immune pathway signatures - transcriptomic data from 703 melanoma tumour samples from Leeds melanoma cohort (see methods for details)

Table S5 – Pathway enrichment analysis from RNAseq of cell line inducible overexpression system for *PPP2R3B* (analysis excludes *PPP2R3B*), highlights suppression of the unfolded protein response and endoplasmic reticulum (ER) protein folding - comparison of pre- and post-induction samples in triplicate, at 6 and 16 hours post induction (see methods for details).

Table S6 - Reverse Phase Protein Array (RPPA) raw data from cell line inducible overexpression system for *PPP2R3B* demonstrates enrichment for mammalian target of rapamycin (mTOR) and hypoxia-induced factor 1 (HIF-1) signalling pathways, with prominent biological signatures of response to heat and stress - comparison of pre- and post-induction samples in triplicate, at 6 and 16 hours post-induction (see methods for details).

Table S7 - RNAseq data from cell line inducible overexpression system for *PPP2R3B*, significantly (Padj<0.05) differentially expressed genes in both cell lines in triplicate, at 6 and 16 hours post-induction (see methods for details). *C21orf91* was identified as the most significantly differentially expressed in both cell lines at both time points, other than *PPP2R3B*.

Supplementary

Melanoma Transcriptomic Data – Upregulated Pathways

The genes positively correlated with PPP2R3B are predominantly enriched in non-immune pathwaysm, consistent with the lack of association between PPP2R3B expression and TILs or any specific immune cell score. There are only 24 pathways significantly enriched in these genes at FDR<0.05 (Table below).

Using undirected interactions, the nodal genes in a network putting together all these pathways are: MAPK11 (betweenness=2237) and PLCB3 (betweenness=1120).

With directed interactions, the most important genes are MAPK11 (betweenness=103) and NFATC1 (betweenness=59).

Supplementary Table 3 - Melanoma Transcriptomic Data – Upregulated Pathways

**Rank**	**Pathway**	**FDR**
1	Rap1 signaling pathway(K)	6.10E-04
2	Axon guidance(K)	6.10E-04
3	ECM-receptor interaction(K)	9.94E-04
4	PI3K-Akt signaling pathway(K)	3.49E-03
5	DNA methylation(R)	5.66E-03
6	Pathways in cancer(K)	9.73E-03
7	Beta1 integrin cell surface interactions(N)	0.0157
8	Oxidative stress response(P)	0.0209
9	Arf1 pathway(N)	0.0281
10	Ras signaling pathway(K)	0.0281
11	Hedgehog signaling events mediated by Gli proteins(N)	0.0281
12	NoRC negatively regulates rRNA expression(R)	0.0281
13	EPH-Ephrin signaling(R)	0.0281
14	RNA Polymerase I Transcription(R)	0.0308
15	Signaling by VEGF(R)	0.035
16	MAPK signaling pathway(K)	0.0388
17	Focal adhesion(K)	0.0388
18	Endocytosis(K)	0.0388
19	Extracellular matrix organization(R)	0.0388
20	Shigellosis(K)	0.0463
21	EPHA forward signaling(N)	0.0463
22	DNA replication(P)	0.0498
23	Beta-catenin independent WNT signaling(R)	0.0498
24	Cell junction organization(R)	0.0528
25	Hedgehog signaling pathway(P)	0.054

Melanoma Transcriptomic Data – Downregulated Pathways

The genes negatively correlated with *PPP2R3B* expression are predominantly enriched in immune pathways. There are 80 pathways, the Table below shows the top 25.

Using undirected interactions, the top 5 nodal genes in a network putting together all these pathways are: TGFB1 (betweenness=8395) and AURKA (betweenness=5133) and CD8A (betweenness=4893), RAC2 (4880) and LCK (betweenness=4855).

With directed interactions, the most important genes are LCK (betweenness=174), HLA-G (betweenness=110), MYD88 (betweenness=104), TGFB1 (betweenness=86) and CD8A (betweenness=82).

Supplementary Table 4 - Melanoma Transcriptomic Data – Downregulated Pathways

**Rank**	**Pathway**	**FDR**
1	Natural killer cell mediated cytotoxicity(K)	3.50E-08
2	Antigen processing and presentation(K)	2.33E-07
3	Cytokine-cytokine receptor interaction(K)	2.33E-07
4	Immunoregulatory interactions between a Lymphoid and a non-Lymphoid cell(R)	5.45E-07
5	IL12-mediated signaling events(N)	6.57E-07
6	Malaria(K)	6.57E-07
7	Retinoid metabolism and transport(R)	6.57E-07
8	Graft-versus-host disease(K)	1.17E-06
9	IL12 signaling mediated by STAT4(N)	1.28E-06
10	Regulation of Insulin-like Growth Factor (IGF) transport and uptake by Insulin-like Growth Factor Binding Proteins (IGFBPs)(R)	1.81E-06
11	Cell adhesion molecules (CAMs)(K)	3.05E-06
12	Post-translational protein phosphorylation(R)	1.77E-05
13	Chemokine signaling pathway(K)	3.29E-04
14	G alpha (i) signalling events(R)	3.29E-04
15	Primary immunodeficiency(K)	4.49E-04
16	IL23-mediated signaling events(N)	4.49E-04
17	Osteoclast differentiation(K)	6.46E-04
18	Response to elevated platelet cytosolic Ca2+(R)	8.66E-04
19	Hematopoietic cell lineage(K)	1.02E-03
20	Th17 cell differentiation(K)	2.12E-03
21	TCR signaling in naive CD4+ T cells(N)	2.12E-03
22	African trypanosomiasis(K)	2.32E-03
23	TCR signaling in naive CD8+ T cells(N)	3.26E-03
24	Leishmaniasis(K)	3.28E-03
25	Extrinsic prothrombin activation pathway(B)	3.54E-03
